# Residue-specific binding of Ni(II) ions influences the structure and aggregation of amyloid beta (Aβ) peptides

**DOI:** 10.1038/s41598-023-29901-5

**Published:** 2023-02-27

**Authors:** Elina Berntsson, Faraz Vosough, Teodor Svantesson, Jonathan Pansieri, Igor A. Iashchishyn, Lucija Ostojić, Xiaolin Dong, Suman Paul, Jüri Jarvet, Per M. Roos, Andreas Barth, Ludmilla A. Morozova-Roche, Astrid Gräslund, Sebastian K. T. S. Wärmländer

**Affiliations:** 1 Department of Biochemistry and Biophysics, Arrhenius Laboratories, Stockholm University, 106 91 Stockholm, Sweden; 2 Department of Chemistry and Biotechnology, Tallinn University of Technology, Tallinn, Estonia; 3 Department of Medical Biochemistry and Biophysics, Umeå University, 901 87 Umeå, Sweden; 4 The National Institute of Chemical Physics and Biophysics, Tallinn, Estonia; 5 Institute of Environmental Medicine, Karolinska Institutet, Nobels Väg 13, 171 77 Stockholm, Sweden; 6 Department of Clinical Physiology, Capio St. Göran Hospital, St. Göransplan 1, 112 19 Stockholm, Sweden; 7 Chemistry Section, Arrhenius Laboratories, Stockholm University, 106 91, Stockholm, Sweden

**Keywords:** Neuroscience, Metals, Peptides, Protein folding, Biochemistry, Biophysical chemistry, Metals, Neurochemistry, Peptides, Protein folding, Proteins, Biophysics, Biological fluorescence, Protein folding, Neurological disorders

## Abstract

Alzheimer’s disease (AD) is the most common cause of dementia worldwide. AD brains display deposits of insoluble amyloid plaques consisting mainly of aggregated amyloid-β (Aβ) peptides, and Aβ oligomers are likely a toxic species in AD pathology. AD patients display altered metal homeostasis, and AD plaques show elevated concentrations of metals such as Cu, Fe, and Zn. Yet, the metal chemistry in AD pathology remains unclear. Ni(II) ions are known to interact with Aβ peptides, but the nature and effects of such interactions are unknown. Here, we use numerous biophysical methods—mainly spectroscopy and imaging techniques—to characterize Aβ/Ni(II) interactions in vitro, for different Aβ variants: Aβ(1–40), Aβ(1–40)(H6A, H13A, H14A), Aβ(4–40), and Aβ(1–42). We show for the first time that Ni(II) ions display specific binding to the N-terminal segment of full-length Aβ monomers. Equimolar amounts of Ni(II) ions retard Aβ aggregation and direct it towards non-structured aggregates. The His6, His13, and His14 residues are implicated as binding ligands, and the Ni(II)·Aβ binding affinity is in the low µM range. The redox-active Ni(II) ions induce formation of dityrosine cross-links via redox chemistry, thereby creating covalent Aβ dimers. In aqueous buffer Ni(II) ions promote formation of beta sheet structure in Aβ monomers, while in a membrane-mimicking environment (SDS micelles) coil–coil helix interactions appear to be induced. For SDS-stabilized Aβ oligomers, Ni(II) ions direct the oligomers towards larger sizes and more diverse (heterogeneous) populations. All of these structural rearrangements may be relevant for the Aβ aggregation processes that are involved in AD brain pathology.

## Introduction

Alzheimer’s disease (AD), the leading cause of dementia worldwide, is a progressive, irreversible, and currently incurable chronic neurodegenerative disorder^1–3^, primarily manifesting as short-term memory loss. Pathological hallmarks of AD include brain atrophy, with extensive brain deposits of amyloid plaques and neurofibrillary Tau tangles occurring years before symptom manifestation^3–5^. The plaques, which consist mainly of amyloid-β (Aβ) peptides aggregated into insoluble fibrils^6^, display a characteristic cross-β structure at the core of their constituent fibrils^7,8^. The plaques are the end-product of an aggregation process involving formation of extra- and intracellular intermediates such as neurotoxic Aβ oligomers^9–14^. The oligomeric aggregates may spread from neuron to neuron via exosomes^15,16^. However, the relationship between Aβ aggregation, neurodegenerative mechanisms, cognitive decline, the proposed amyloid cascade hypothesis, and disease progression is not fully understood^2,13,14,17^.

The 36–43 residues long Aβ peptides found in the plaques are produced by enzymatic cleavage of the membrane-binding amyloid-β precursor protein, APP^18^. In monomeric form, the Aβ peptides are intrinsically disordered and soluble in water. The central and C-terminal segments are hydrophobic and may interact with membranes or fold into a hairpin conformation that likely is required for aggregation^19^. The negatively charged N-terminal segment is hydrophilic and readily interacts with metal ions and other cationic molecules^20–23^.

AD brains typically display altered metal homeostasis^17,24,25^, and AD plaques accumulate metals such as calcium (Ca), copper (Cu), iron (Fe), and zinc (Zn)^26–28^. Thus, dysregulated metal chemistry might be part of the AD pathology process^29–32^. The precursor protein APP is known to bind Cu and Zn ions^33^, and a possible physiological role of APP (and perhaps its fragments) might be to regulate the Cu(II) and Zn(II) concentrations in the neuronal synaptic clefts, where these ions are released in their free form^34^ and where Aβ aggregation may be initiated^35^. Metal ions such as Cu(II), Fe(II), Mn(II), Pb(IV) and Zn(II) have previously been shown to bind to specific Aβ residues and modulate the Aβ aggregation pathways^20,29,36–40^. Binding of metal ions, and also of other cationic molecules such as polyamines, has furthermore been reported to modulate and sometimes inhibit Aβ toxicity^21,22,41^. However, it is unclear which possible metal interactions may be relevant for AD pathology, and which exogenous or endogenous metal ions may participate in such interactions^30–32^.

Nickel (Ni) is a common metal in the industrialized world, where it is used in e.g. stainless steel alloys, Ni–Cd batteries, coins, and jewelry. As a result of low-level exposure, between 10 and 20% of all people have developed some degree of contact allergy towards Ni^42,43^. It is therefore important to clarify the health effects of long-term Ni exposure, including potential effects on neurodegenerative diseases^44^. Some studies have demonstrated specific binding between Ni(II) ions and N-terminal Aβ fragments, with possible effects on Aβ structure and toxicity^41,45–47^. Yet, the interactions between pathologically relevant (i.e., full-length) Aβ peptides and Ni ions are poorly explored.

In this study, we use a range of biophysical spectroscopy and imaging techniques to investigate in vitro interactions between Ni(II) ions and Aβ peptides, with a focus on characterizing binding properties and effects on Aβ structure and aggregation. The different Aβ peptides studied include the pathologically relevant Aβ(1–40), Aβ(4–40), and Aβ(1–42) variants, together with the Aβ(1–40)(H6A, H13A, H14A) mutant. Because the Aβ peptides interact with membranes^48^, and as membrane-disruption is a possible toxicity mechanism for Aβ oligomers^30^, the measurements have been carried out in aqueous solution as well as in a membrane-mimetic model consisting of micelles of the SDS (sodium dodecyl sulfate) detergent^49^. The results are compared to previous studies of the effects of both different chemical environments and metal ion interactions on Aβ peptides^37,38,40,48–53^.

### Biological relevance of Ni and sources of exposure

Even though Ni function is limited in human biology^54^, it has been suggested as an essential element in humans^55^, just as it is for many human-associated bacteria^56^ and possibly all higher plants^57^. Elevated Ni concentrations are however toxic to plants^58^. In animals, inadequate Ni amounts have shown adverse effects on nutrient absorption and metabolism^59,60^. Due to the abundance of Ni in many plant-based foods^58,61^, Ni deficiency is unlikely to occur in humans, even though only some 10% of ingested Ni is absorbed^62^. Ni concentrations in potable water vary between 2 and 13 mg/L with a WHO limit of 70 mg/L, which is exceeded in Ni mining regions, where Ni concentrations of 200 mg/L have been found^63^. Respiratory Ni exposure is related to Ni industries and fossil fuel combustion, which overall are the main global sources of Ni emissions^58,62^, and to cigarette smoking including e-cigarettes^38,64^. In addition to Ni–Cd batteries, the main industrial uses of Ni is as a whitening agent in Cu alloys for e.g. coins and jewellery, which may cause allergic contact dermatitis^58^, and as a provider of corrosion resistance in steel alloys for e.g. surgical tools, biomedical implants, and body piercings. One study of Ni-containing hip prosthetic devices found that Ni blood concentration rose about twofold after metal-on-metal hip arthroplasty^65^. Ni is also present in some formulations for dental amalgam fillings^66^.

The human health risks of Ni exposure are well-known and widespread, as Ni use and exposure has gradually grown from human prehistory^67^ to modern times^68^. Ni is known to be haematotoxic, immunotoxic, neurotoxic, genotoxic, reproductive toxic, pulmonary toxic, nephrotoxic, hepatotoxic and carcinogenic^58,69^. Other pathological effects of Ni exposure in the occupational settings are rhinitis, asthma, nasal septum perforation, nasal sinus cancer, and respiratory cancer^70^. Ni can pass the placental barriers and accumulate in the fetus^71^. It can also pass the blood–brain barrier, and brain accumulation can result from high levels of exposure^54^. Yet, most people experience low exposure levels^66^, and the most common health effect is allergic reactions^42,43^.

The neurotoxic properties of Ni are well documented, but data on Ni in neurodegenerative disorders are scarce^44^. A case report of skin tissue Ni concentrations in a patient recovering from ALS after metal chelation described a Ni concentration of 850 µg/kg^72^. A 44 year old ALS patient died after 9 years of heavy metal exposure in a nickel–cadmium battery factory^73^. Increased blood Ni concentrations have been detected in multiple sclerosis (MS) patients^74^, and soil Ni was found to be elevated in a Canadian MS cluster^75^. Recent data also indicate a possible contribution from Ni in the causation of neurodevelopmental dysfunctional states such as autism^76^.

As a component of tobacco, cigarette smoke, and air pollution, Ni may contribute to environmental risk factors for AD. Mice exposed to a Ni nanoparticle model of air pollution showed doubled brain levels of Aβ_40_ and Aβ_42_ within 24 h, even at a permissible limit of nickel hydroxide exposure according to occupational safety and health standards^77^. Another study reported higher yet not statistically significantly elevated Ni concentrations in post-mortem brain and ventricular fluid of AD patients (n = 14), compared to healthy controls (n = 15)^25^. A recent study reported that Ni(II) ions interfered with aggregation of the Tau protein^78^.

## Materials and methods

### Samples and preparations

Ni(II) acetate and 2-(N-Morpholino)ethanesulfonic acid hydrate (MES) buffer were purchased from Sigma (Sigma/Merck KGaA, Darmstadt, Germany). The SDS detergent was bought from ICN Biomedicals Inc (USA). Sodium chloride and sodium hydroxide were purchased from Sigma-Aldrich (St. Louis, MO, USA).

Wild-type (wt) Aβ(1–42) peptides, abbreviated as Aβ_42_, with the primary sequence DAEFR_5_HDSGY_10_EVHHQ_15_KLVFF_20_AEDVG_25_SNKGA_30_IIGLM_35_VGGVV_40_IA, were purchased synthetically manufactured from JPT Peptide Technologies (Germany), while recombinantly produced Aβ_42_ peptides were purchased from rPeptide LLC (USA). Recombinantly produced wild-type (wt) Aβ(1–40) peptides, abbreviated as Aβ_40_, as well as N-terminal truncated Aβ(4–40) peptides, were purchased as lyophilized powder from AlexoTech AB (Umeå, Sweden). The Aβ_40_ peptides were either unlabeled, uniformly ^15^N-labeled, or uniformly ^13^C,^15^N-labeled. A recombinantly produced mutant version of Aβ_40_, where the three histidine residues H6, H13, and H14 have been replaced with alanines, i.e. Aβ(1–40)(H6A, H13A, H14A) was also purchased from AlexoTech AB. This mutant is here abbreviated as Aβ_40_(NoHis). All Aβ variants were stored at − 80 °C until use, when they were dissolved to monomeric form before the measurements. The Aβ_40_ and Aβ(4–40) peptides were then dissolved in 10 mM NaOH to 100 µM concentration, and sonicated for 5 min in an ice-bath to dissolve possible pre-formed aggregates. Finally, buffer was added to the peptide solutions. All preparation steps were performed on ice, and the peptide concentrations were determined by weighing the dry powder and/or by NanoDrop measurements of dissolved material.

### Preparation of Aβ_42_ oligomers

Monomeric solutions of Aβ_42_ peptides were prepared via size exclusion chromatography, according to the following procedure. First, lyophilized Aβ_42_ powder (1 mg) was dissolved in pure dimethyl sulfoxide (DMSO; 250 µL). A solution of 5 mM NaOH (pH = 12.3) was used to equilibrate a Sephadex G-250 HiTrap desalting column (GE Healthcare, Uppsala), which was then washed with 10–15 mL of 5 mM NaOD (pD = 12.7)^79^. The Aβ_42_ solution in DMSO was added to the column, followed by 5 mM NaOD (1.25 mL). Peptide fractions in 5 mM NaOD were then collected on ice at a flow rate of 1 mg/mL. Ten fractions of 1 mL were collected in 1.5 mL low-binding reaction tubes. The Aβ_42_ concentration in each fraction was measured with a NanoDrop instrument (Eppendorf, Germany) at 280 nm, using a molar extinction coefficient of 1280 M^−1^ cm^−1^ for the single tyrosine residue in the peptide^80^. Liquid nitrogen was used to flash-freeze the fractions, which then were topped with argon gas, and stored at − 80 °C until use. Two well-defined sizes of SDS-stabilized Aβ_42_ oligomers—named according to the SDS concentration used: AβO_0.05%SDS_ (approximately dodecamers) and AβO_0.2%SDS_ (approximately tetramers)—were prepared using an established protocol^81^ with the following modifications: the preparations were carried out in D_2_O without the original dilution step, and at a fourfold lower peptide concentration^82^. The reaction mixtures, consisting of 100 µM Aβ_42_ peptide in phosphate buffered saline (PBS) buffer containing either 0.05% SDS or 0.2% SDS, which corresponds to 1.7 mM and 6.9 mM SDS, respectively, were incubated at 37 °C for 24 h together with 0–500 µM of Ni(II) acetate. Liquid nitrogen was used to flash-freeze the prepared oligomer solutions, which then were stored at − 20 °C until further use. When thawed at room temperature for experimental analyses, the oligomers were stable for several days.

### NMR spectroscopy measurements of Aβ_40_ binding to Ni(II) ions

1D and 2D nuclear magnetic resonance (NMR) spectra were recorded on Bruker Avance 500 and 700 MHz spectrometers equipped with cryoprobes. First 42 μM and then 84 μM of Ni(II) acetate was added to 84 μM of monomeric Aβ_40_ peptides, either ^13^C,^15^N-double-labelled or ^15^N-mono-labelled, in 20 mM sodium phosphate buffer at pH 7.3 or pH 5.6 (90/10 H_2_O/D_2_O). During the titrations, 2D ^1^H,^15^N-HSQC and 2D ^1^H,^13^C-HSQC spectra were recorded at 5 °C. Measurements were also conducted in the presence of 50 mM SDS detergent, at 25 °C. As the critical micelle concentration for SDS is 8.2 mM in water at 25 °C^83^, micelles have clearly formed under these conditions. SDS micelles are simple membrane models suitable for NMR spectroscopy due to their small size, i.e. on average 62 molecules per micelle^49^. Thus, there is approximately 0.8 mM of SDS micelles in the sample, i.e., around 10× more micelles than Aβ_40_ peptides, which means that no micelle should harbour multiple Aβ peptides. All NMR data were processed and evaluated using the Topspin software (v. 3.2), employing already published HSQC crosspeak assignments for Aβ_40_ in buffer^84–86^ and in the presence of SDS micelles^51^.

### CD spectroscopy measurements of Ni(II)-induced changes in Aβ secondary structure

Circular dichroism (CD) was carried out in a Chirascan CD spectrometer (Applied Photophysics Ltd., U.K.) using a 2 mm quartz cuvette containing 600 µl of Aβ peptide in 20 mM phosphate buffer, pH 7.3. The studied Aβ variants were Aβ_40_ (10 µM), Aβ_40_(NoHis) (10 µM), and Aβ(4–40) (5 µM).

Measurements were conducted either in buffer only or with added SDS micelles. CD spectra were recorded at 25 °C between 190 and 260 nm, using steps of 0.5 nm. After the first recorded spectrum, 50 mM SDS detergent was added to some of the samples. Then, small volumes of Ni(II) acetate (2 mM and 10 mM stock solutions) were titrated to each sample, in steps of 2 µM, 4 µM, 16 µM, 56 µM, 156 µM, 256 µM, and finally 512 µM. All data was processed with an eight points smoothing filter (Savitsky-Golay) using the Chirascan Pro-Data v.4.4.1 software (Applied Photophysics Ltd., U.K.).

### Binding affinity of Ni(II)·Aβ complexes

Binding affinities for Ni(II)·Aβ complexes were estimated by fitting NMR and CD titration data, respectively, to Eq. (1) (the Morrison equation^87^):

1 $$I={I}_{0}+\frac{{I}_{\infty }-{I}_{0}}{2*\left[A\beta \right]}*\left(\left({K}_{D}+\left[Ni\right]+\left[A\beta \right]\right)-\sqrt{{\left({K}_{D}+\left[Ni\right]+\left[A\beta \right]\right)}^{2}-4*\left[Ni\right]*\left[A\beta \right]}\right)$$

This equation assumes a single metal binding site, where [Aβ] is the peptide concentration, [Ni] is the concentration of the titrated Ni(II) ions, I_0_ is the initial signal intensity, I_∞_ is the steady-state (saturated) signal intensity at the end of the titration series, and K_D_ is the dissociation constant for the Ni(II)·Aβ complex. As no corrections for buffer conditions are done, the computed dissociation constants should be considered as apparent (K_D_^App^).

For the CD data, a binding curve was generated by plotting the CD intensity at 208 nm vs the Ni(II) concentration. For the NMR data, binding curves were generated by plotting HSQC crosspeak intensities versus Ni(II) concentration. The measured crosspeak intensities were normalized to the intensity of the V40 crosspeak for each step of added Ni(II) ions.

### Aβ_40_ aggregation kinetics monitored via ThT fluorescence measurements

The kinetics of amyloid aggregation over time for Aβ_40_ peptides together with Ni(II) ions was monitored via measurements with a 96-well plate reader (FLUOstar Omega) of the fluorescence signal of the dye Thioflavin T (ThT), a molecular probe that displays strong fluorescence intensity when bound to amyloid material^88,89^. The samples contained 10 μΜ Aβ_40_ peptide, 10 mM sodium phosphate buffer, pH 7.4, 40 μΜ ThT dye, and 0, 1, 2.5, 5, 7.5, 10, 20, or 50 μM of Ni(II) acetate. The peptide concentration was determined using a NanoDrop microvolume spectrophotometer. Measurements were recorded at three-minute intervals for 24 h at 37 °C, with five replicate samples per condition and excitation and emission wavelengths of 440 and 480 nm, respectively. The samples were continuously shaken (orbital mode) between the measurements. To determine the maximum growth rate and the half-time of Aβ aggregation, the resulting ThT fluorescence data were fitted to a sigmoidal curve using Eq. (2)^90^:

2 $$F\left( t \right) = F_{0} + \frac{A}{{1 + \exp \left[ { - r_{\max } \left( {t - t_{1/2} } \right)} \right]}}$$

where F_0_ is the baseline fluorescence intensity, A is the total increase in fluorescence intensity, r_max_ is the maximum growth rate, and t_1/2_ is the time when half of the Aβ monomers have aggregated.

### Atomic force microscopy images of Aβ_40_ aggregates

Images of Aβ_40_ aggregates were recorded with a BioScope Catalyst (Bruker Corp., USA) atomic force microscope (AFM), operating in peak force mode in air and using MSLN and SLN cantilevers (Bruker Corp., USA). The scan rate was 0.51 Hz, with a resolution of 512 Å ~ 512 pixels. Samples of 10 μΜ Aβ_40_ peptide were incubated for 24 h with either 0, 1, 10, or 50 μΜ Ni(II) acetate, using the same conditions as in the ThT experiments described above. At the end of the procedure, 30 μL samples were diluted in 30 μL Milli-Q water and then applied on freshly prepared mica substrates. After 20 min, the mica substrates were washed three times with Milli-Q water and left to air-dry.

### Blue native polyacrylamide gel electrophoresis of Aβ_42_ oligomers

The Aβ_42_ oligomer samples prepared with 0–500 µM Ni(II) acetate, as described in the materials section, were analyzed with blue native polyacrylamide gel electrophoresis (BN-PAGE) using the Invitrogen electrophoresis system. First, 4–16% Bis–Tris Novex gels (ThermoFisher Scientific, USA) were loaded with Aβ_42_ oligomer samples (10 μL) in addition to the Amersham High Molecular Weight Calibration Kit for native electrophoresis (GE Healthcare, USA). The gels were run at 4 °C according to the Invitrogen instructions (ThermoFisher Scientific, USA). Staining was done with the Pierce Silver Staining Kit (ThermoFisher Scientific, USA).

### Infrared spectroscopy

Fourier-transformed infrared (FTIR) spectra of the Aβ_42_ oligomers prepared with 0–500 µM Ni(II) acetate, as described in the materials section, were recorded on a Tensor 37 FTIR spectrometer (Bruker Optics, Germany) operating in transmission mode at room temperature and equipped with a liquid nitrogen-cooled MeCdTe detector and a sample shutter. During the measurements, the instrument was continuously purged with dry air. 8–10 µL of the 80 µM Aβ_42_ oligomer samples were placed between two flat CaF_2_ discs, which were separated by a 50 µm plastic spacer that had been covered with vacuum grease at the periphery. The mounted IR cuvette was put in a holder inside the sample chamber, and was then allowed to sit for at least 20 min after the chamber lid was closed, to remove H_2_O vapor. FTIR spectra were recorded between 1900 and 800 cm^−1^, at a resolution of 2 cm^−1^ and with 6 mm aperture. The IR intensity above 2200 cm^−1^ was blocked with a germanium filter, and that below 1500 cm^−1^ with a cellulose membrane, to increase the light intensity in the relevant spectral range^91^. The OPUS 5.5 software was used for analysis and plotting of the spectra. Second derivatives were computed with a smoothing factor of 17.

### Fluorescence measurements of Ni(II)-induced formation of dityrosine in Aβ_40_

A Jobin Yvon Horiba Fluorolog 3 fluorescence spectrometer (Longjumeau, France) was used to record fluorescence emission spectra between 330 and 500 nm (excitation at 315 nm) at room temperature of 10 µM Aβ_40_ peptide dissolved in 20 mM MES buffer, pH 7.3. The samples were put in a quartz cuvette with 4 mm path length (volume 1 mL). To investigate the effect of Ni(II) ions on dityrosine formation, one sample contained 100 µM Ni(II) acetate. The control sample contained 50 µM of the chelator EDTA, to remove possible free metal ions. Spectra were recorded after 0 and 6 h of incubation, where the sample was kept at room temperature without agitation or other treatment. All experiments were conducted in triplicate, and before the final measurement, 300 µM of EDTA was added to the sample to remove metal ions.

## Results

### NMR spectroscopy: molecular details of Ni(II) binding to the Aβ_40_ monomer

**Figure 1:**
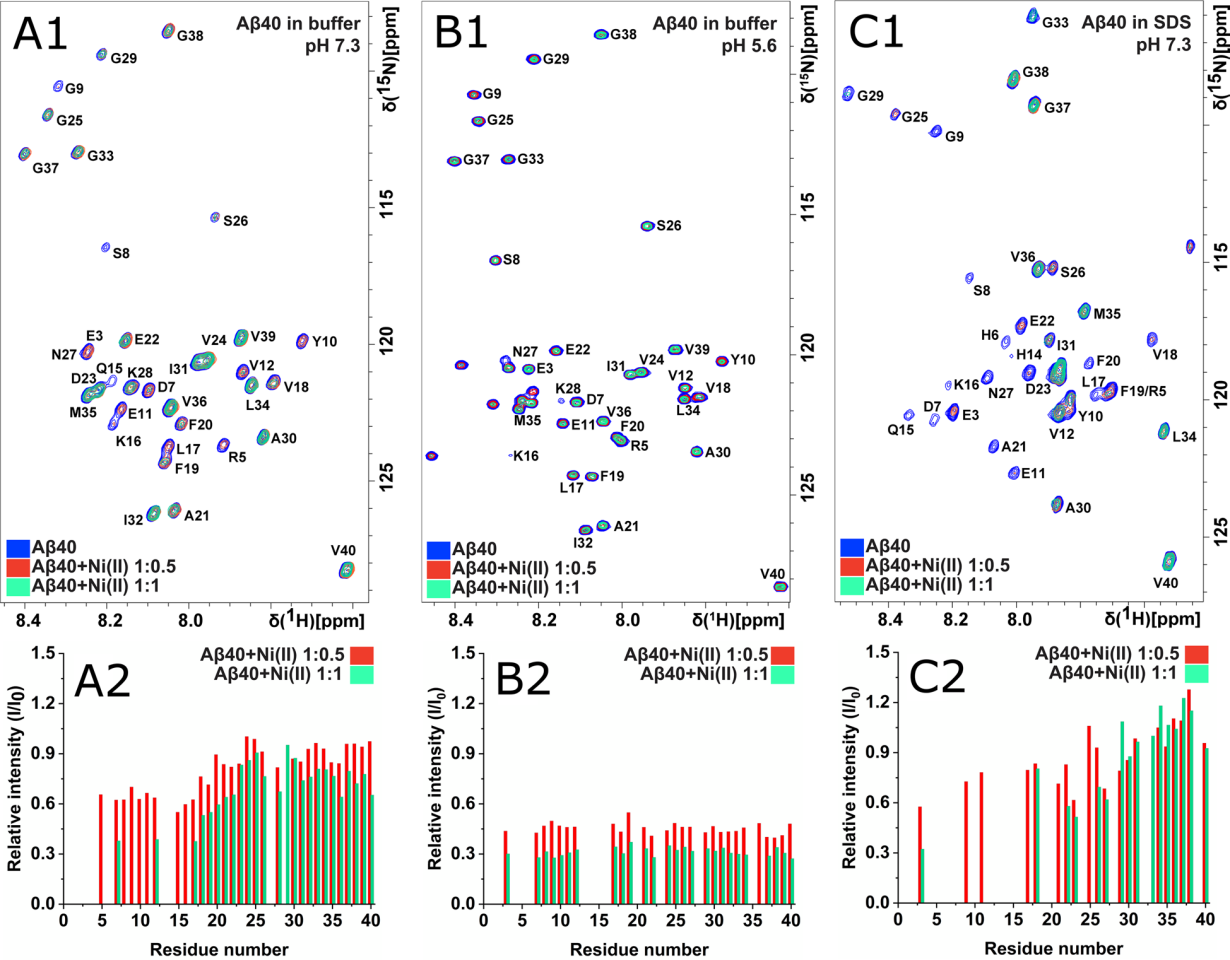
NMR 2D ^1^H,^15^N-HSQC-spectra of 84 µM ^15^N-labeled Aβ_40_ peptides before (blue peaks) and after addition of first 42 µM (red peaks) and then 84 µM (teal peaks) Ni(II) acetate. Spectra were recorded at 5 °C in 20 mM sodium phosphate buffer at pH 7.3 (**A**), at pH 5.6 (**B**), and at pH 7.3 together with 50 mM SDS detergent (**C**). The peak intensity in the bar charts is given as the ratio between the crosspeak intensity with added Ni(II) ions relative to the intensity before addition of Ni(II) ions, i.e. I/I_0_.

**Figure 2:**
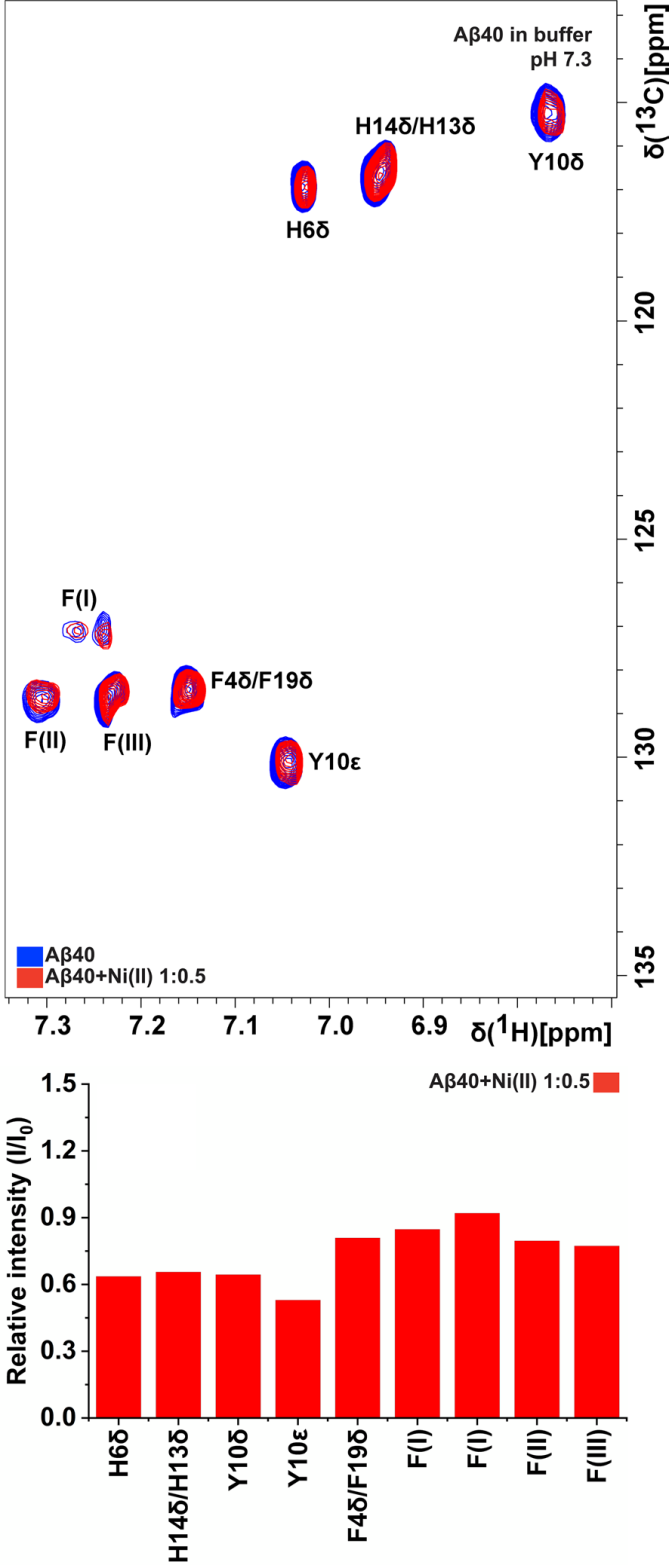
NMR 2D ^1^H,^13^C-HSQC spectra of 84 µM ^13^C,^15^N-labeled Aβ_40_ peptides in 20 mM phosphate buffer, pH 7.3, before (blue) and after (red) addition of 42 µM Ni(II) acetate. Some Phe crosspeaks could not be assigned to individual residues, and are instead listed as F(I), F(II), and F(III).

High-resolution NMR experiments were conducted to investigate possible residue-specific molecular interactions between Ni(II) ions and monomeric Aβ_40_ peptides. Figures 1 and 2 show 2D ^1^H,^15^N-HSQC spectra for the amide crosspeak region for 84 µM ^13^C,^15^N-labeled Aβ_40_ peptides at either pH 7.3 or pH 5.6, recorded before and after addition of first 42 µM and then 84 µM Ni(II) acetate, i.e. Ni(II):Aβ_40_ ratios of respectively 1:2 and 1:1. Similar measurements were conducted also for Aβ_40_ peptides together with SDS micelles (50 mM SDS detergent concentration) at pH 7.3 (Fig. 1C).

For Aβ_40_ in aqueous pH 7.3 buffer, addition of Ni(II) ions induces a concentration-dependent loss of amide crosspeak intensity, especially in the N-terminal region (Fig. 1A). This indicates specific binding of Ni(II) ions to N-terminal Aβ_40_ residues. The specific loss of N-terminal crosspeak intensity is likely caused by intermediate or even slow (on the NMR time-scale) chemical exchange between a free and a bound state of the Aβ_40_ peptides, similar to the effect induced by Cu(II) and Zn(II) ions^40,92^, probably together with paramagnetic quenching effects of the Ni(II) ions^93^. When the Ni(II) ions are added to the sample, no new crosspeaks corresponding to Ni(II)-bound Aβ_40_ peptides are observed (Fig. 1A). This suggests that no single well-defined Ni(II)·Aβ_40_ complex exists. Instead, a range of Ni(II)-bound states of the Aβ_40_ peptides are likely present, probably at different stages of aggregation and oligomerization. Each state is then too weakly populated to create distinct NMR crosspeaks. This is in line with earlier NMR studies reporting that Aβ_40_ peptides are in a dynamic exchange between NMR-observable monomers and heterogeneous NMR-invisible oligomers (a “dark state”)^92,94^.

There is also a general loss of crosspeak intensity for all Aβ_40_ residues, including the central and C-terminal amino acids, upon addition of Ni(II) acetate. This effect is likely caused by a combination of non-specific Ni(II) binding interactions, on an intermediate or slow NMR time-scale, and by the Ni(II) ions promoting aggregation of the Aβ_40_ peptides into complexes that are too large to be observed with HSQC NMR, or they may simply precipitate out of the solution.

At pH 5.6, the loss of crosspeak intensity after added Ni(II) ions is very uniform, i.e., there is no residue-specific binding (Fig. 1B). The main difference at this lower pH is that histidine residues are protonated, as they have pKa values around 6.8 in short peptides^95^. Naturally, protonated His residues are less prone to bind cationic metal ions. The loss of residue-specific Ni(II) ion binding at pH 5.6 therefore strongly indicates that histidines are involved in the observed residue-specific Ni(II) ion binding at neutral pH. This is supported by the NMR results at pH 7.3 for the Aβ_40_ aromatic side chains, where the aromatic rings of the N-terminal residues His6, His13, and His14, together with Tyr10, display a somewhat larger loss of crosspeak signal intensity than the aromatic rings of the Phe4, Phe19, and Phe20 residues (Fig. 2).

Specific loss of crosspeak intensity for N-terminal residues, upon addition of Ni(II) acetate, is observed also for Aβ_40_ peptides positioned in SDS micelles (Fig. 1C), which constitute a simple model for bio-membranes^48,49^. The central and C-terminal Aβ regions are known to insert themselves as α-helices into SDS micelles^48,51^, and thus, the ^1^H,^15^N-HSQC spectrum for Aβ_40_ in SDS micelles corresponds to an α-helical conformation of the Aβ peptide. The N-terminal segment is known to remain unstructured outside the micelle surface, where it is available for interactions with e.g. metal ions^96^. The C-terminal residues are unaffected by the added Ni(II) ions (Fig. 1C), which shows that they are neither affected by direct Ni(II)-binding, nor by Ni(II)-induced aggregation—most likely Aβ peptides cannot aggregate when bound to SDS micelles, at least when there is on average less than one peptide per micelle. Thus, also in the presence of SDS micelles (Fig. 1C), the NMR spectrum reflects Ni(II) ion binding to monomeric Aβ_40_ peptides.

### CD spectroscopy measurements of Aβ secondary structure

**Figure 3:**
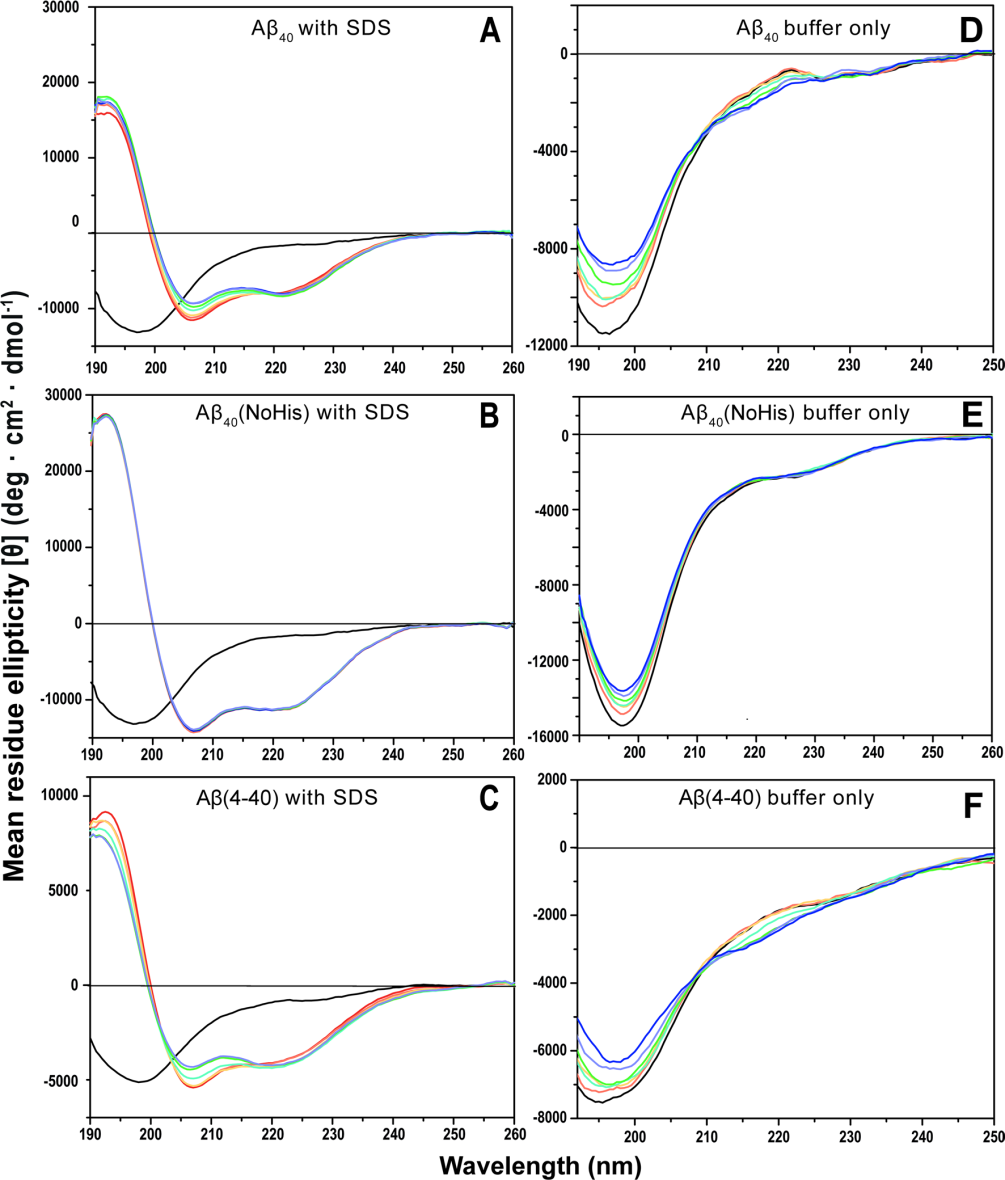
CD spectra of Ni(II) acetate titrated to Aβ peptides in 20 mM phosphate buffer, pH 7.3, at 25 °C. The titrations were conducted either in the presence of SDS micelles (**A**–**C**) or in aqueous buffer only (**D**–**F**), for three different peptide variants, i.e. 10 µM Aβ_40_ (**A**,**D**), 10 µM Aβ_40_(NoHis) (**B**,**E**), and 5 µM Aβ(4–40) (**C**,**F**). The black spectra show Aβ peptides in buffer only. For samples A–C, 50 mM SDS was then added (red spectra). Next, for all samples, Ni(II) acetate was titrated in steps of 2 µM (orange), 4 µM (yellow), 16 µM (turquoise), 56 µM (green), 156 µM (purple), and finally 256 µM (blue spectra).

CD spectroscopy was used to investigate possible changes in Aβ secondary structure upon addition of Ni(II) ions, both in aqueous buffer and in a membrane-mimetic environment (i.e., SDS micelles). The three peptide variants Aβ_40_, Aβ_40_(NoHis) mutant, and Aβ(4–40) were investigated. In aqueous buffer, the CD spectra for monomers of all three variants display typical random coil signals with minima around 196–198 nm (Fig. 3).

Addition of Ni(II) ions to Aβ peptides in aqueous buffer induces concentration-dependent changes in the CD spectra for the monomers of all three peptide variants (Fig. 3D–F). For the Aβ_40_(NoHis) peptide, these changes correspond to a decrease in intensity, without changing the shape of the spectrum (Fig. 3). This likely means that the Ni(II) ions induce peptide aggregation and precipitation, thereby reducing the effective Aβ concentration in the solution.

For the Aβ_40_ and Aβ(4–40) variants, addition of Ni(II) ions induces structural transitions between two distinct conformations, as evidenced by the isodichroic points around 210 nm (Fig. 3D,F). Aβ peptides in solution are known to contain some polyproline II (PPII) helix structure, especially at low temperatures^50^. The loss of signal intensity around 196–198 nm, and the isodichroic points around 210 nm, might be compatible with a conversion of PPII helix into random coil structure^50,97,98^. However, the difference spectra created by subtracting the CD spectra with no added Ni(II) acetate from those with 256 μM Ni(II) acetate, shown in Supp. Fig. S1, clearly correspond to β-sheet secondary structure^99^. Formation of β-sheets upon addition of Ni(II) ions is supported also by the IR spectra shown in Supp. Fig. S2. We therefore conclude that Ni(II) ions induce β-sheet structure in Aβ_40_ and Aβ(4–40) peptides, in aqueous solution at neutral pH.

When SDS micelles were added to the three peptide variants, all of them adopted α-helical secondary structures, producing CD signals with characteristic minima around 208 and 222 nm (Fig. 3A–C). This is consistent with previous studies reporting that Aβ peptides adopt α-helical conformations in membrane-like environments, at least when there is on average less than one peptide per micelle^48,49,51,96,100^. The Aβ_40_(NoHis) variant, where the three His residues have been replaced with alanines, displays the strongest α-helical CD signal after addition of SDS, compared to the intensity of the Aβ_40_(NoHis) random coil signal before adding SDS (Fig. 3). This is reasonable given the strong propensity of alanines to form α-helices^101^. The Aβ(4–40) peptide also shows a relatively strong α-helical CD signal, compared to the Aβ(4–40) random coil intensity before added SDS. This might be related to a lack of α-helical structure^51^ in the first three residues of Aβ_40_, which are missing in the Aβ(4–40) variant.

Addition of Ni(II) ions induces a concentration-dependent loss of CD signal around 208 nm, but not around 222 nm, for the Aβ_40_ and the Aβ(4–40) peptides, although not for the Aβ_40_(NoHis) mutant (Fig. 3). As the 222 nm signal intensity remains approximately constant, the observed spectral changes do not correspond to a general loss of α-helicity but may rather be due to a change in helix supercoiling, i.e. when two or more α-helices form coiled coils via hydrophobic interactions^102–104^. The degree of helix supercoiling is known to be reflected by the [θ_222_]/[θ_208_] ratio, where ratios close to 1 reflect large amounts of superhelicity^105^. During the titrations with Ni(II) acetate the [θ_222_]/[θ_208_] ratio increases from 0.70 to 0.89 for Aβ_40_, and from 0.75 to 1.03 for the Aβ(4–40) variant (Table 1). In both cases this would correspond to a significant increase in superhelicity. Such metal-induced changes of Aβ superhelicity, in a membrane environment, has previously been reported to be induced by Cu(II) ions^96^. The lack of any Ni(II)-induced structural effects in the Aβ_40_(NoHis) mutant (Fig. 3; Table 1) appears to support Ni(II) binding to Aβ via the His residues. But it is also possible that the His residues are necessary for forming a coiled-coil Aβ structure.

**Table 1:** CD signal intensities at 208 nm and 222 nm for the three Aβ variants Aβ_40_, Aβ_40_(NoHis), and Aβ(4–40), as a function of added Ni(II) acetate.

	Wavelength (nm)	0 µM Ni(II)	2 µM Ni(II)	4 µM Ni(II)	16 µM Ni(II)	56 µM Ni(II)	156 µM Ni(II)	256 µM Ni(II)
Aβ_40_	208	− 11,131	− 10,808	− 10,557	− 9928	− 9515	− 9064	− 9025
	222	− 7793	− 7925	− 8074	− 8241	− 8339	− 8012	− 8114
	222/208	0.700	0.733	0.765	0.830	0.8764	0.884	0.899
Aβ_40_(NoHis)	208	− 14,062.7	− 13,979	− 14,112.3	− 13,919.9	− 13,962.9	19,802.9	13,725.2
	222	− 11,184.4	− 11,100.6	− 11,249.3	− 11,138.9	− 11,172.6	− 11,149.8	− 11,039.3
	222/208	0.7953	0.7941	0.7971	0.8002	0.8002	0.8078	0.8043
Aβ(4–40)	208	− 5335.7	− 5265.9	− 5258.7	− 4876.8	− 4338.2	− 4235.2	− 4150.7
	222	− 3978.0	− 3989.9	− 4193.3	− 4302.0	− 4205.2	− 4176.1	4268.9
	222/208	0.746	0.758	0.797	0.882	0.969	0.986	1.028

### Estimates of Aβ·Ni(II) binding affinity

**Figure 4:**
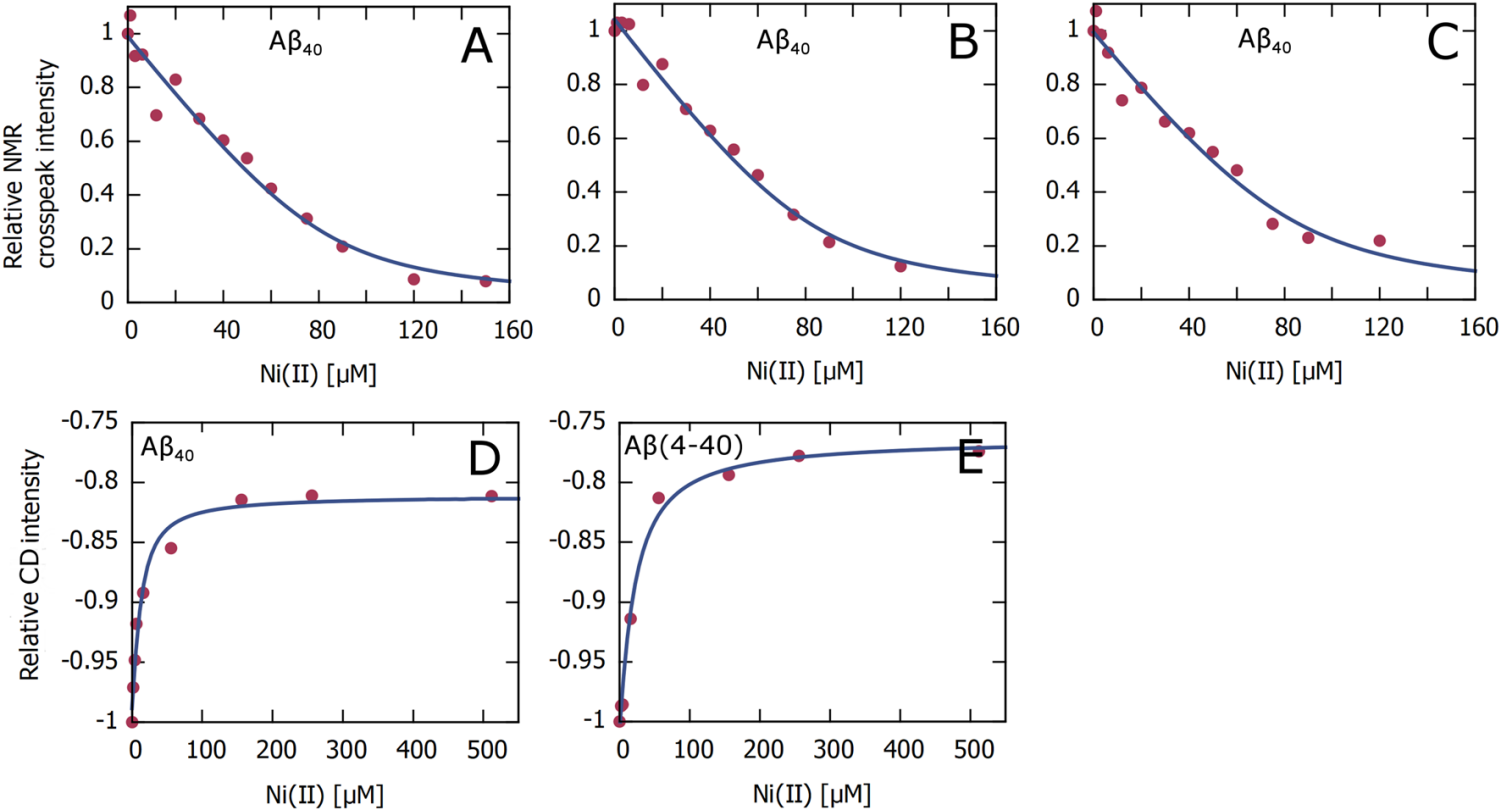
Binding curves for Ni(II)·Aβ complexes, derived from the NMR data in Fig. 1A and the CD data in Fig. 3A,C, respectively. The NMR and CD signal intensities are given as the ratio between the intensity with added Ni(II) ions relative to the intensity before addition of Ni(II) ions, i.e. I/I0. Apparent binding affinities were obtained by fitting Eq. (1) to the curves. (**A**–**C**) NMR crosspeak intensity vs Ni(II) concentration for three of the crosspeaks/residues in Fig. 1A, i.e. for 84 µM Aβ_40_ in 20 mM sodium phosphate buffer, pH 7.3 at 5 °C. (**D**,**E**) CD intensity at 208 nm vs Ni(II) concentration for the CD data in Fig. 3A,C, i.e. for 10 µM Aβ_40_ (**D**) and 5 µM Aβ(4–40) (**E**) in 20 mM phosphate buffer, pH 7.3 at 25 °C.

Ni(II)·Aβ binding curves were generated by plotting crosspeak intensities from the NMR titrations (Fig. 1A) and 208 nm intensities from the CD titrations (Table 1) versus Ni(II) concentration (Fig. 4). Fitting Eq. (1) to these curves yields apparent dissociation constants (K_D_) for the Ni(II)·Aβ complexes.

For the CD data (Fig. 4D,E), the signal intensities have been normalized to the first value in each titration series, i.e. the signal intensity without added Ni(II) ions. The derived K_D_ values are 7.8 µM for binding to Aβ_40_, and 17.2 µM for binding to the Aβ(4–40) variant. These K_D_ values should however only be considered as approximations, as there may not be a direct correlation between Ni(II) binding and the structural changes observed in the CD spectra (Fig. 3). Our NMR measurements (Fig. 1C) confirm earlier studies showing that the N-terminal Aβ segment is free to interact with metal ions also when the central and C-terminal Aβ segments are inserted into SDS micelles^48,51,96^. Yet, earlier studies with Cu(II) ions have shown that the binding affinity for metal ions is reduced when the central Aβ segment is bound to some other entity^106^, although this effect appears to be minor for SDS micelles^96^.

For the NMR data, each crosspeak in Fig. 1A generates one binding curve. However, the Ni(II)-induced loss of crosspeak intensity is not only related to concentration-dependent chemical exchange, on an intermediate NMR time-scale, but also to Ni(II)-induced peptide aggregation and paramagnetic quenching of the NMR signal. Even though these effects are somewhat mitigated by normalizing the crosspeak intensity values to the V40 crosspeak intensity, for each titration step (thereby obtaining the relative intensity scale used in Fig. 4A–C), the derived K_D_ values should only be considered as rough approximations. The binding curves shown in Fig. 4A–C correspond to the three Aβ_40_ crosspeaks that display the strongest apparent binding, with K_D_ values of respectively 5.3 µM, 6.7 µM, and 7.0 µM. These values are very similar to the apparent K_D_ value of 7.8 µM derived for Aβ_40_ from the CD measurements (Fig. 4D), but they should still only be regarded as lower limits for the true K_D_ value. Thus, we conclude that the binding affinity for the Aβ_40_·Ni(II) complex is in the low µM range.

### Effects of Ni(II) ions on Aβ_40_ aggregation kinetics

**Figure 5:**
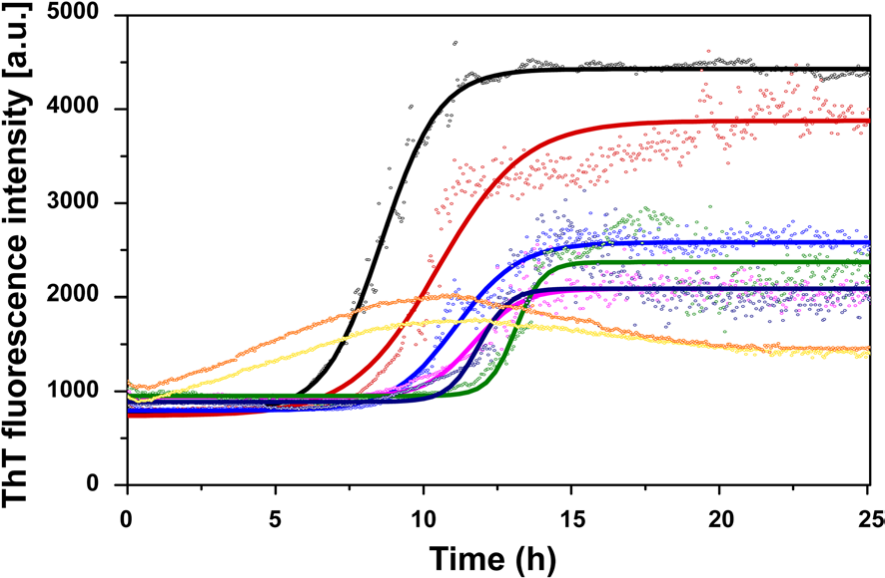
ThT kinetic time curves for aggregation of 10 µM Aβ_40_, in 10 mM sodium phosphate buffer, pH 7.4, together with different concentrations of Ni(II) acetate: 0 µM (black), 1 µM (red), 2.5 µM (azure blue), 5 µM (magenta), 7.5 µM (green), 10 µM (navy blue), 20 µM (yellow), and 50 µM (orange). The solid lines show curves fitted with Eq. (2) to the ThT data sets.

To investigate the influence of Ni(II) ions on Aβ_40_ aggregation, samples of 10 µM Aβ_40_ were incubated for 24 h in the absence or presence of increasing concentrations of Ni(II) acetate. The resulting ThT curves are shown in Fig. 5, and the r_max_, t_1/2_, and A parameters obtained from fitting the curves to Eq. (2) are shown in Table 2. For sub-stoichiometric Ni(II):Aβ_40_ ratios, the Ni(II) ions slow down the Aβ_40_ aggregation kinetics in a concentration-dependent manner. The aggregation half-time (t_1/2_) increases with increasing Ni(II) concentrations, i.e. from around 8 h without Ni(II) ions to over 13 h with 10 µM Ni(II) ions. The maximum aggregation rate r_max_ shows no systematic change with increasing Ni(II) concentration, instead it fluctuates around 0.9 h^−1^ to 1 h^−1^ (Table 2). The end-point ThT fluorescence intensities (parameter “A” in Eq. 2) generally decrease with increasing Ni(II) concentrations (Fig. 5), suggesting that less amyloid material (ThT-binding aggregates) is formed when Ni(II) ions are present. Other explanations are however possible, such as binding competition between ThT molecules and Ni(II) ions, or formation of very large Aβ aggregates that may block the transmitted light, or simply precipitate out of the solution. For the samples with high Ni(II) concentrations, i.e. 20 µM and 50 µM, above the stoichiometric Ni(II):Aβ_40_ ratio, the ThT curves first increase but then decrease back towards the starting value (Fig. 5). Our best explanation for this unusual behaviour is formation of large samples that precipitate, thereby effectively reducing the Aβ_40_ concentration in the sample. It was not possible to fit Eq. (2) to these data curves.

**Table 2:** Parameters r_max,_ t_1/2_, and A for aggregation of 10 µM Aβ_40_ in the presence of different concentrations of Ni(II) acetate.

Ni(II)	0 µM	1 µM	2.5 µM	5 µM	7.5 µM	10 µM	20 µM	50 µM
r_max_ [h^−1^]	0.96 ± 0.22	1.08 ± 0.36	0.88 ± 0.34	1.0 ± 0.45	1.02 ± 0.85	0.96 ± 0.58	n/a	n/a
t_1/2_ [h]	8.3 ± 1.5	10.2 ± 0.3	11.8 ± 1.2	11.2 ± 0.7	12.5 ± 0.5	13.5 ± 1.4	n/a	n/a
A	4115 ± 436	2967 ± 238	2085 ± 575	1449 ± 480	1511 ± 132	1803 ± 442	n/a	n/a

The parameters were obtained from sigmoidal curve-fitting (Eq. 2) to the ThT curves shown in Fig. 5.

### AFM imaging: effects of Ni(II) ions on the morphology of Aβ_40_ aggregates

**Figure 6:**
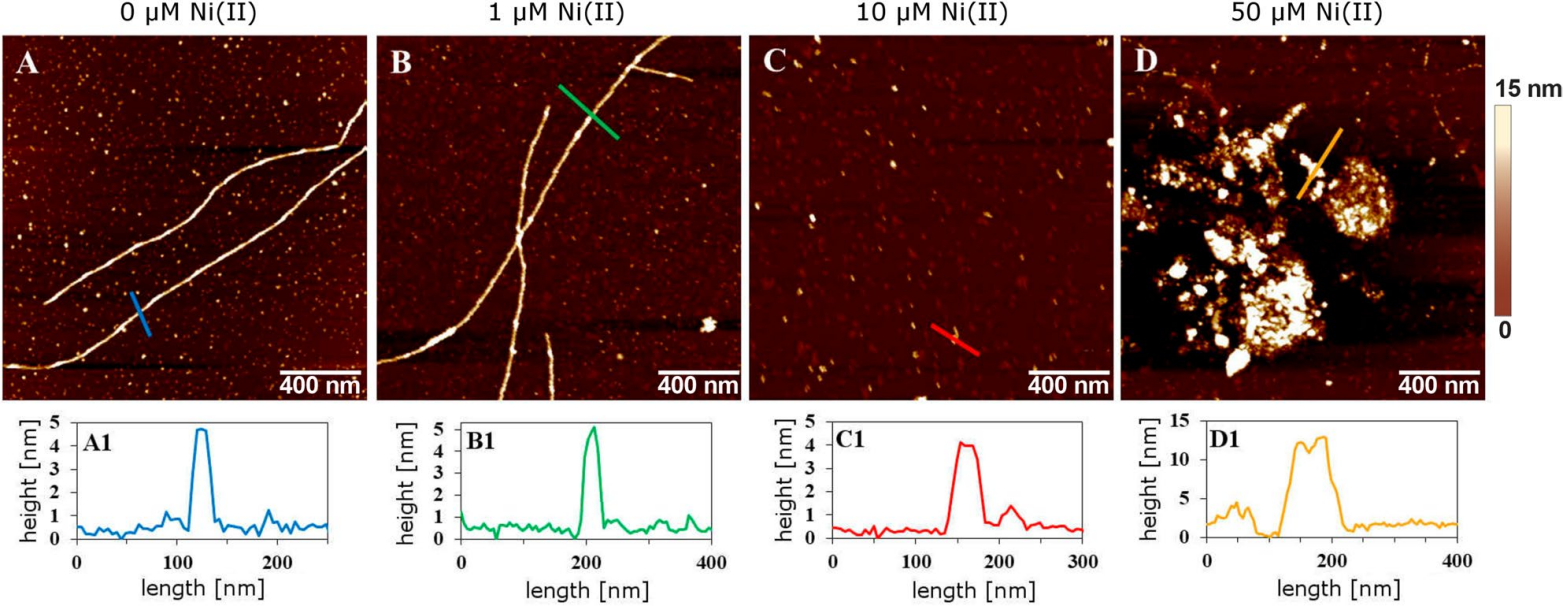
Top row: AFM images of the aggregation products obtained after incubation of 10 µM Aβ_40_ peptides for 24 h together with either 0, 1, 10, or 50 µM Ni(II) acetate. Bottom row: Representative AFM cross-sections of respectively Aβ_40_ amyloid fibrils (**A**,**B**) and Aβ_40_ unstructured aggregates (**C**,**D**), corresponding to the colored lines shown in the AFM images.

To further characterize the influence of Ni(II) ions on Aβ_40_ fibril morphology, AFM images were recorded on Aβ_40_ aggregates formed after 24 h incubation in the presence and the absence of Ni(II) (Fig. 6). Without Ni(II) acetate, 10 µM Aβ_40_ formed typical amyloid fibrils with an apparent height around 4–5 nm (Fig. 2A), which is in line with previously published work on Aβ fibrils formed in vitro^107–109^. A similar apparent height was observed in the presence of 1 µM Ni(II) ions (Fig. 6B). The presence of 10 µM Ni(II) ions, i.e. a 1:1 Ni(II):Aβ_40_ ratio, significantly reduces fibril formation: only occasional very short Aβ_40_ fibril fragments were observed, which display the same height as the fibrils formed by Aβ_40_ alone (Fig. 6C). In the presence of 50 µM Ni(II) ions no fibrils were present, but instead amorphous clumps of Aβ_40_ aggregates with variable heights around 13 nm (Fig. 6D). These results are consistent with the concentration-dependent effects of Ni(II) acetate on the Aβ_40_ aggregation process observed with the ThT measurements (Fig. 5).

### Influence of Ni(II) ions on Aβ_42_ oligomer formation

**Figure 7:**
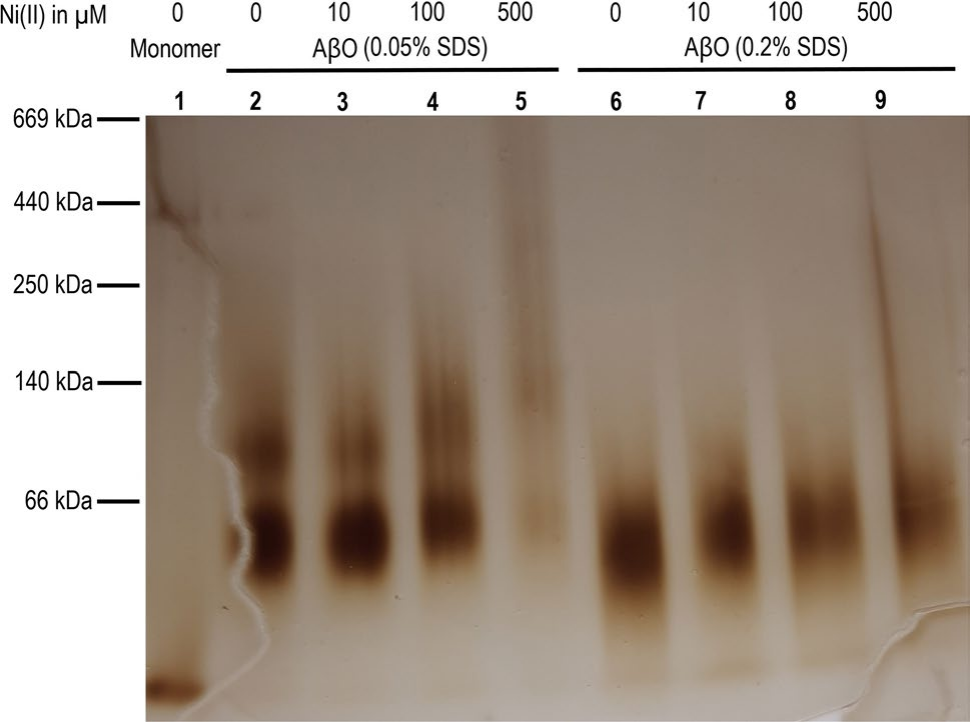
Effects of Ni(II) ions on formation of SDS-stabilized Aβ_42_ oligomers (AβO_0.05%SDS_ and AβO_0.2%SDS_) studied by BN-PAGE. Lane 1: Monomers; Lanes 2–5: AβO_0.05%SDS_ oligomers with respectively 0, 10, 100, and 500 µM Ni(II) ions; Lanes 6–9: AβO_0.2%SDS_ oligomers with respectively 0, 10, 100, and 500 µM Ni(II) ions.

PAGE analysis was used to investigate the effect of Ni(II) ions on the formation of Aβ_42_ oligomers, using a previously published protocol for formation of stable and homogeneous Aβ_42_ oligomers together with SDS detergent^81,82^. While most of the current study investigates variants of the Aβ_40_ peptide, oligomers of Aβ_40_ are not stable and therefore not suitable model systems. Thus, SEC-purified monomeric solutions of synthetic Aβ_42_ peptides were mixed with low concentrations of SDS, i.e., below the critical micelle concentration. Incubation of Aβ_42_ with 0.2% SDS (6.9 mM) leads to formation of mostly tetrameric oligomers (AβO_0.2%SDS_), while incubation with 0.05% SDS (1.7 mM) produces larger oligomers—predominantly dodecamers (AβO_0.05%SDS_)^81^ (Fig. 7, lanes 2 and 6). Figure 7 also shows the effect of different Ni(II) concentrations on oligomer formation. In the absence of Ni(II) ions, the AβO_0.05%SDS_ (Lane 2) and AβO_0.2%SDS_ (Lane 6) oligomers are the dominating species in their respective lanes. With increasing Ni(II) concentration, the band intensity for the major oligomeric structure declines in both cases, while smears towards higher molecular weights appear (Fig. 7, Lanes 3–5 and 7–9). Formation of AβO_0.05%SDS_ is largely disrupted when Ni(II) ions are present at 500 μM concentration (Aβ_42_:Ni(II) molar ratio of 1:5), while the smear extends over almost the entire length of the lane (Fig. 7, Lane 5). A similar, but less drastic effect is observed for the smaller AβO_0.05%SDS_ (Fig. 7, Lane 9). For both types of SDS-stabilized oligomers, Ni(II) ion concentrations above ~ 100 μM disrupt oligomer formation and more heterogeneous Aβ_42_ oligomeric solutions containing larger oligomers are produced. A similar smearing effect of Ni(II) ions on the formation of SDS-stabilized Aβ_42_ oligomers was observed also by SDS-PAGE experiments. These results are shown and discussed in the supplementary information (Fig. S3).

### FTIR spectroscopy of Aβ_42_ oligomers formed in the presence of Ni(II) ions

**Figure 8:**
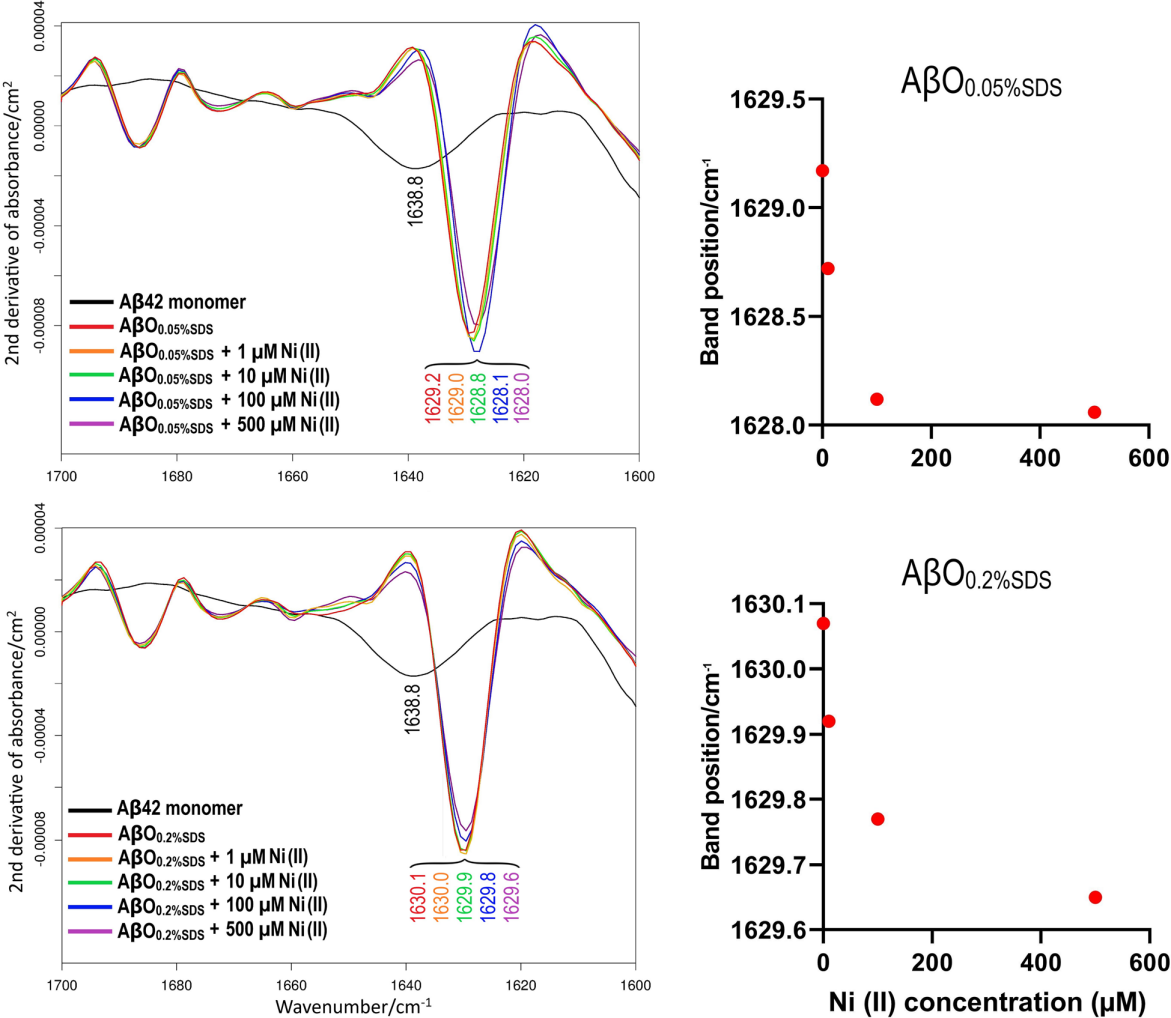
Transmission FTIR data for synthetic AβO_0.05%SDS_ (upper row) and synthetic AβO_0.2%SDS_ (lower row) formed in the presence of different concentrations of Ni(II) ions. The spectra show raw data without normalization. Left: Second derivatives of IR absorbance spectra in the amide I range (1700–1600 cm^−1^) at zero Ni(II) (red); 1 μM Ni(II) (orange); 10 μM Ni(II)—(green); 100 μM Ni(II) (blue); 500 μM Ni(II) (violet). The black spectrum is for Aβ_42_ monomers. Right: Dependence on Ni(II) ion concentration for the position (in cm^−1^) of the main amide I band. For a more clear presentation, the data point at 1 µM was omitted.

FTIR spectroscopy is a powerful technique for studying the secondary structure of proteins^110–116^, and can be used to characterize the backbone conformation for different aggregation states of amyloid proteins, including Aβ peptides^117–119^. Here, the effects of Ni(II) ions on the secondary structures of both AβO_0.05%SDS_ and AβO_0.2%SDS_ oligomers were studied with transmission mode FTIR spectroscopy, using a D_2_O-based buffer. The results are presented in Fig. 8 as second derivatives of IR absorption spectra, where negative bands indicate the component bands of the absorption spectra. The respective absorbance spectra are shown in Fig. S4 of the Supplementary Information.

For both types of Aβ_42_ oligomers, two bands are resolved in the amide I region (i.e., 1700–1600 cm^−1^): a high intensity, low wavenumber band around 1630 cm^−1^ (the main band for β-sheet structure), and a low intensity, high wavenumber band at 1685 cm^−1^. This pattern with a split double-band in the amide I region is routinely considered as indicative of the anti-parallel β-sheet structure^117,118,120^. When Ni(II) acetate is introduced during the Aβ_42_ oligomer formation reactions, the main band is slightly down-shifted: for AβO_0.05%SDS_ (Fig. 8, upper row) from 1629.2 cm^−1^ in the absence of Ni(II) acetate to 1628.0 cm^−1^ at 500 μM of Ni(II) acetate (the highest concentration), and for AβO_0.2%SDS_ (Fig. 8, lower row) from 1630.1 to 1629.6 cm^−1^ upon addition of Ni(II) acetate up to 500 μM. The downshift is smaller for the oligomers prepared at the higher SDS concentration, indicating that they are less sensitive to Ni(II)-induced effects on the oligomer conformation. The spectral changes observed with Ni(II) are in interesting contrast to the absence of spectral effects upon addition of Li(I) ions^121^. The downshift is mainly observed in the presence of Ni(II) concentrations between 10 and 100 μM, which agrees with the binding affinities estimated from the CD and NMR results. Detailed analysis of the spectra in Fig. 8 shows that the shifts in band position are associated with a widening of the main β-sheet band on its low wavenumber side, indicating a higher abundance of larger oligomers^82^. Most of this widening occurs between 10 and 100 μM Ni(II). It further increases between 100 and 500 μM Ni(II), which correlates with the appearance of a high molecular weight smear on the BN-PAGE gel (Fig. 7).

Our previous study on the IR characterization of Aβ_42_ oligomers has revealed a relationship between oligomer size and position of the main band in the amide I region^82^. According to these findings, the downshift of the main IR band is associated with an increase in average oligomer size and a concomitant extension of their β-sheet structure. However, the Ni(II)-induced size change is rather modest. The band position of AβO_0.2%SDS_ at the highest Ni(II) ion concentration is still higher than the AβO_0.05%SDS_ band position in the absence of Ni(II) ions, indicating that the oligomers contain less than twelve peptides. Also, the band position of AβO_0.05%SDS_ at 500 µM Ni(II) concentration is considerably higher than that of oligomers formed in the absence of SDS (1623.1 cm^−1^), which had an average molecular weight of ~ 100 kDa according to Western blotting^82^. Thus, the dominant β-sheet containing oligomer species of AβO_0.05%SDS_ seem to be smaller than ~ 100 kDa.

The interpretation of the current IR results is in good agreement with the results from the gel electrophoresis experiments, particularly with the BN-PAGE data (Fig. 7). Both IR and PAGE results indicate that addition of Ni(II) ions appears to interfere with the SDS-induced conversion of Aβ_42_ monomers into homogeneous and stable oligomeric structures, instead favoring formation of larger and more diverse (heterogeneous) oligomer populations.

### Ni(II)-induced dityrosine formation

**Figure 9:**
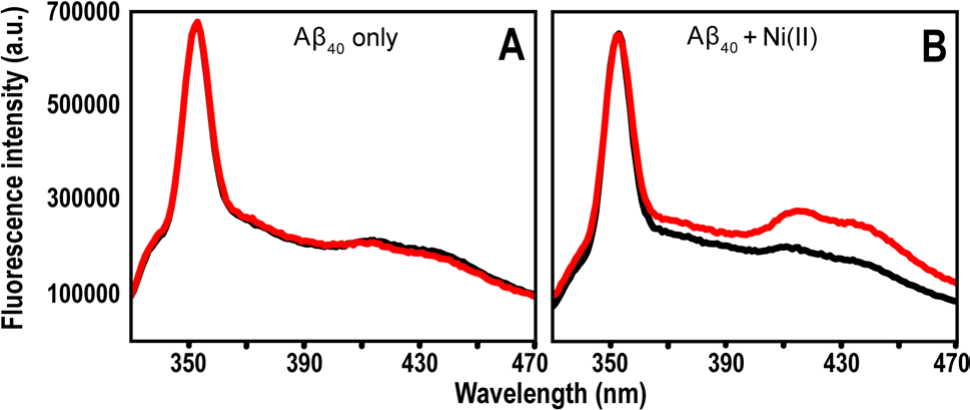
Fluorescence spectra of 10 µM Aβ_40_ in 20 mM MES buffer, pH 7.3, incubated together with 50 µM EDTA (**A**) or with 100 µM Ni(II) acetate (**B**). Black line—0 h; red line—6 h.

Fluorescence measurements were carried out to investigate if binding of Ni(II) ions could induce formation of covalent dityrosine crosslinks in Aβ peptides, similar to what has been observed for Cu(II) ions^122–125^. Fluorescence emission spectra for Aβ_40_ peptides incubated over time with or without Ni(II) acetate are shown in Fig. 9.

The control sample without added Ni(II) ions, which contained 50 µM EDTA to ensure no free metal ions were present, displayed virtually identical spectra before and after 6 h of incubation (Fig. 9A). This is in stark contrast to the sample with 100 µM Ni(II) ions added, where two large new peaks around 410 nm and 435 nm formed during the incubation time (Fig. 9B). The peak around 410 nm is from dityrosine^126^, while the peak around 435 nm likely is from a different but related system, such as excimers^127^. These results are not surprising, as nickel is known to be very redox-active. Both the Ni(I)/Ni(II) and Ni(II)/Ni(III) redox pairs could be involved in generating the oxygen radicals required for dityrosine formation^128^. It should be noted that weak peaks around 410 nm and 435 nm are present in both samples already at time zero (Fig. 9). This shows that some dityrosine cross-links have been generated even before the experiment was initiated, which is consistent with previous reports stating that Aβ peptides can induce oxidative stress on their own, especially in somewhat aggregated states^129,130^.

## Discussion

Nickel is a well-known neurotoxicant, but its role in neurodegenerative diseases remains unclear^44^. Several studies have investigated the possible effects of transition metals such as Cu and Zn in AD neuropathology, with an emphasis on interactions with the amyloid-forming Aβ peptides^29–32^. We therefore interpret our current results on Aβ interactions with Ni(II) ions mainly in the light of earlier work on Aβ binding to Cu(II) and Zn(II) ions.

### Residue-specific binding of Ni(II) ions to Aβ peptides

Our NMR results show that equimolar amounts of Ni(II) ions display residue-specific binding to the N-terminal segment of the Aβ_40_ peptide (Fig. 1). This is in line with earlier studies showing that Ni(II) ions can bind N-terminal Aβ fragments^45–47^. The 2D ^1^H-^13^C-HSQC NMR data (Fig. 2) suggest that the three histidine residues His6, His13, and His14 are involved as binding ligands, possibly together with the Tyr10 residue. Previous work has established the metal-binding capacity of the Tyr phenol ring^131^, and in Aβ peptides the Tyr10 residue seems to be involved in binding to Pb(IV) ions^38^. The weaker Ni(II)/Aβ_40_ interactions observed at low pH (Fig. 1) further support the histidines being binding ligands, as these residues become protonated at low pH and therefore less prone to interact with cations^92,106,132^. The CD spectroscopy measurements also support the histidines being involved as binding ligands: addition of Ni(II) ions induces structural changes in Aβ_40_ peptides, but not in Aβ_40_(NoHis) mutant peptides (Fig. 3). This indicates that Ni(II) ions do not bind Aβ peptides when the His residues are absent, which is not surprising, given that Ni(II) ions are known to bind His residues such as those in protein His-tags^133^. Thus, Ni(II) ions seem to belong to a family of metal ions that coordinate to the Aβ N-terminal segment mainly by the His residues, just like Ag(I), Cu(II), Fe(II), Hg(II), Mn(II), Zn(II), and possibly Pb(IV) ions^20,37,38,40,47,52,53,92,134,135^. The exact binding coordination could not be determined from our measurements, and it is possible that multiple alternating binding conformations exist, as has been shown for Cu(II) ions^136^.

According to the Irving-Williams series^137^, the binding affinities of certain divalent metal ions to peptides and proteins should follow the order Mn(II) < Fe(II) < Co(II) < Ni(II) < Cu(II) > Zn(II). Metal binding affinities are however notoriously difficult to quantify, as they tend to vary both with the experimental conditions (buffer, temperature) and the employed measurement technique^138^. For example, binding affinities varying by several orders of magnitude have previously been reported for the Aβ·Cu(II) complex, with a consensus value in the low nM region for buffer-corrected affinity^138^. In our earlier studies, we have reported apparent (not buffer-corrected) K_D_ values around 50–100 µM for Mn(II) ions in phosphate buffer, pH 7.35^37^, around 1–10 µM for Zn(II) ions in phosphate or Hepes buffer, pH 7.2^40^, and around 0.5 – 2.5 µM for Cu(II) ions in phosphate or Hepes buffer, pH 7.2–pH 7.35^40,96,106^.

Both the CD and the NMR measurements suggest an affinity in the low µM range for Ni(II) binding to Aβ peptides (Fig. 4), i.e. weaker than Cu(II) ions, stronger than Mn(II) ions, and perhaps somewhat similar to Zn(II) binding affinity, which would be consistent with the Irving-Williams series. As the Ni(II) ions bind to the N-terminal Aβ segment, the binding affinity should be rather the same for Aβ_40_ and Aβ_42_ peptides, and also for shorter Aβ versions such as Aβ(1–28) and Aβ(1–16). The CD measurements indicate that the Ni(II) binding affinity to the truncated Aβ(4–40) peptide is similar to, or even somewhat weaker than, the affinity to the full-length Aβ_40_ peptide (Figs. 3 and 4). This is unexpected, as the Aβ(4–40) peptide has been reported to contain an N-terminal binding motif that supposedly provides very strong binding to Cu(II) and Ni(II) ions^139^, i.e. possibly fM affinity for Cu(II) ions^140^. Binding of metal ions to truncated Aβ variants is biologically relevant as such variants, and especially Aβ(4–42), are abundant in amyloid plaques from both healthy and AD brain tissues^141–144^.

### Effects of Ni(II) ions on Aβ structure and aggregation

Similar to e.g. Ag(I), Cu(II), Hg(II), and Zn(II) ions^39,40,52,53^, Ni(II) ions retard Aβ_40_ amyloid formation in a concentration-dependent manner by directing the aggregation pathways towards non-fibrillar amorphous aggregates as demonstrated both by ThT fluorescence and AFM imaging (Figs. 5 and 6). Already at a 1:1 Ni(II)/Aβ ratio, Aβ_40_ fibrillation appears to be completely inhibited. This supports earlier studies reporting that Ni(II) ions can influence protein aggregation^145^. We have previously shown that low Zn(II) concentrations induce a Zn(II)-bound structure that prevents the Aβ peptides from forming the β-hairpin required for fibrillation^19,40^. At higher Zn(II) concentrations β-sheet structure was induced^92^, similar to our current observations with CD spectroscopy that Ni(II) ions induce β-sheet structure in Aβ_40_ and Aβ(4–40) peptides (Fig. 3D,F). Aβ aggregation is promoted by the direct electrostatic effect of binding cations to the anionic Aβ peptides, thereby reducing repulsion between the Aβ peptides^40^. Given that Ni(II) and Zn(II) ions have similar charge and binding ligands, they likely affect Aβ aggregation and fibrillation via similar mechanisms.

Although Aβ fibrils, such as those shown in Fig. 6A,B, are the end products of Aβ aggregation, intermediate aggregates known as soluble oligomers are now generally considered to be the main toxic species in AD pathology^146,147^. The toxic mechanisms are unclear, but may involve membrane disruption^30^, as some studies have reported that Aβ oligomers can form membrane-spanning “pores” that can induce leakage of e.g. Ca(II) ions^148^. Interestingly, other studies have reported that this harmful Ca(II) leakage can be inhibited by histidine-associating compounds, such as imidazole, Zn(II), and Ni(II) ions^41,149^. Because Cu(II), Zn(II), and other divalent ions have been shown to affect the structure, stability, and/or toxicity of Aβ oligomers^149–152^, it is worth noting that both our FTIR (Figs. 8 and S2) and CD results (Figs. 3 and S1) show that Ni(II) ions induce structural changes in both Aβ oligomers and Aβ monomers. This is in line with earlier studies showing that both SDS-stabilized Aβ_42_ oligomers^81,153,154^, and SDS micelle-bound Aβ monomers^51,96^ contain surface-exposed N-termini which makes it possible for the N-terminal H6, H13, and H14 residues to interact with metal ions. We speculate that Ni(II) ions affect Aβ oligomer structure in similar ways as Cu(II) and Zn(II) ions do, even though the exact mechanisms are not fully understood^30^. Addition of Ni(II) ions reduces the intensity of the NMR crosspeaks for monomeric Aβ_40_ peptides in random coil structure (Fig. 1), showing that this conformation becomes less populated. But no new NMR crosspeaks appear (Fig. 1), which shows that the Ni(II) ions do not bind to form a single well-defined Ni(II)·Aβ complex. Instead, a range of heterogeneous β-sheet-containing structures are induced, which most likely exist in different stages of aggregation. They can be observed in CD and FTIR spectra, but not in NMR spectra^92,94^.

### Effects of Ni(II) ions on Aβ dityrosine formation

Ni(II) ions may affect the Aβ aggregation processes also via formation of reactive oxygen species (ROS). Nickel is well known as a redox-active metal that can adopt a wide range of oxidation states, i.e., from − 1 to + 4^155^. While this can be usefully employed in engineering contexts such as in Ni–Cd batteries, in biological systems it means that Ni can induce oxidative stress, and this may be one of the main mechanisms of Ni toxicity^69^. Our experiments show that addition of Ni(II) ions initiates formation of Aβ_40_ dityrosine cross-links (Fig. 9), which is a common ROS effect. Earlier studies have shown that bound redox-active Cu ions can initiate dityrosine formation, both in Aβ and other peptides and proteins^96,122–125,152,156,157^. It is therefore not surprising that a similar effect is observed for Ni(II) ions, especially as the NMR results indicate that Tyr10 is one of the Ni(II) binding ligands (Fig. 2). As wt Aβ peptides only contain one Tyr residue, i.e. Tyr10, dityrosine formation must involve two Aβ peptides, which then combine into a covalently linked dimer. Such dityrosine-linked Aβ dimers are of biological significance, as they have been found in amyloid plaques in AD brains^158^. As these plaques contain elevated levels of bound redox-active Cu and Fe ions^26,27^, it is likely that the Aβ dityrosine links observed in AD patients are generated by metal-induced ROS. Because dimerization is the first step towards peptide aggregation, dityrosine formation in Aβ peptides is clearly a process that influences aggregation. In vitro studies have shown that dityrosine-linked Aβ dimers undergo rapid aggregation into oligomers that are stable, soluble, and neurotoxic^159^.

## Conclusions

We here show for the first time that Ni(II) ions bind to the N-terminal segment of biologically relevant (i.e., full-length) Aβ peptides. The Ni(II) binding affinity is in the low µM range, with the three N-terminal His residues and possibly Tyr10 involved as binding ligands. At equimolar amounts, Ni(II) ions impede Aβ fibrillation by directing the aggregation towards amorphous aggregates. The redox-active Ni(II) ions induce dityrosine cross-links via redox chemistry, thereby creating covalent Aβ dimers. Ni(II) ions induce structural alterations in Aβ monomers, both in aqueous buffer (formation of beta sheets) and in membrane-mimicking SDS micelles (likely formation of coil-coil helix), and affect also Aβ oligomerization. Although Ni(II) binding to Aβ is somewhat weaker than Cu(II) binding, the two metal ions induce similar effects on Aβ structure and aggregation. Exposure to stochiometric amounts of Ni(II) ions induces formation of heterogeneous Aβ oligomers, which can be observed with CD and IR but not NMR spectroscopy. These oligomers, which are in a dynamic equilibrium with Aβ monomers, may be important contributors to AD brain pathology.

## Supplementary Information

Supplementary Information.

## Data Availability

All data generated or analysed during this study are included in this published article (and its supplementary information files). The raw data for the spectroscopy measurements are available from the corresponding author on reasonable request.
