# Supplementary material for: Residue-specific binding of Ni(II) ions influences the structure and aggregation of amyloid beta (Aβ) peptides
Source: Sci Rep. 2023 Feb 27;13:3341. doi: 10.1038/s41598-023-29901-5 (PMC9971182; doi:10.1038/s41598-023-29901-5)
Supplement: Supplementary file 1 — Supplementary Information. [file 41598_2023_29901_MOESM1_ESM.pdf]

**Supplementary Information**

# Residue-specific binding of Ni(II) ions influences the structure and aggregation of amyloid-beta (Aβ) peptides

Elina Berntsson[1,2,*], Faraz Vosough[1], Teodor Svantesson[1], Jonathan Pansieri[3], Igor A. Iashchishyn[3], Lucija Ostojić[3], Xiaolin Dong[1], Suman Paul[1], Jüri Jarvet[1,4], Per M. Roos[5,6], Andreas Barth[1], Ludmilla Morozova-Roche[3], Astrid Gräslund[1], Sebastian K.T.S. Wärmländer[7,*]

[1] Department of Biochemistry and Biophysics, Arrhenius Laboratories, Stockholm University, 106 91 Stockholm, Sweden.
[2] Department of Chemistry and Biotechnology, Tallinn University of Technology, Estonia.
[3] Department of Medical Biochemistry and Biophysics, Umeå University, SE-901 87 Umeå, Sweden.
[4] The National Institute of Chemical Physics and Biophysics, Tallinn, Estonia.
[5] Institute of Environmental Medicine, Karolinska Institutet, Nobels väg 13, 171 77 Stockholm, Sweden.
[6] Department of Clinical Physiology, Capio St. Göran Hospital, St. Göransplan 1, 112 19 Stockholm, Sweden.
[7] Chemistry Section, Arrhenius Laboratories, Stockholm University, 106 91 Stockholm, Sweden.

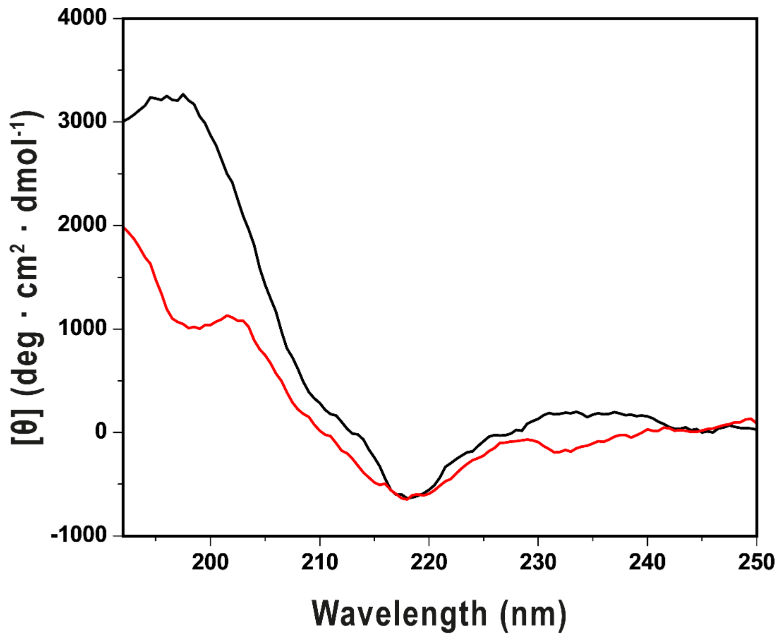


**Fig. S1**. Difference CD spectra created from the data shown in Figs. 3D and 3F, respectively, obtained by subtracting the spectra with no added Ni(II) acetate from the spectra with 256 µM Ni(II) acetate. Black: 10 µM A$\beta_{40}$ wt. Red: 10 µM Aβ(4-40) peptide.

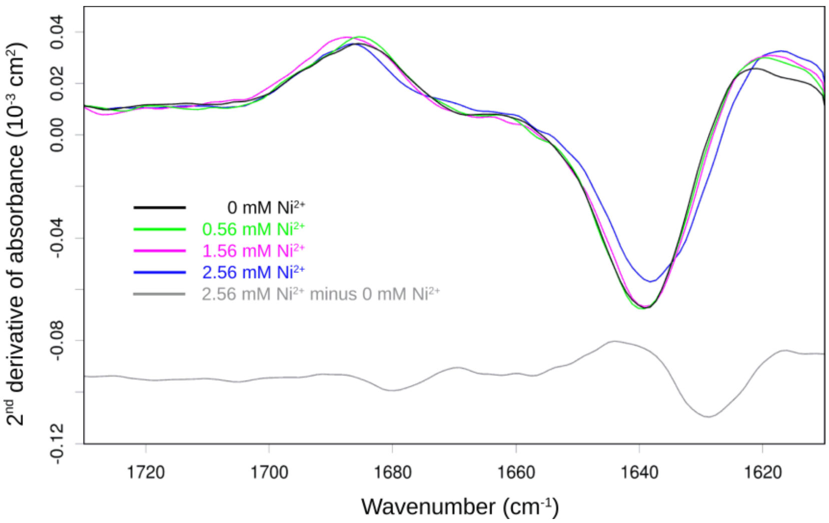


**Fig. S2**. Second derivatives of IR absorbance of 100 µM A$\beta_{40}$ wt peptide at pD 12.0, 0°C, with different Ni(II) concentrations. Band positions are 1638.9 cm$^{-1}$ (zero Ni(II)), 1639.6 cm$^{-1}$ (0.56 mM Ni(II)), 1639.0 cm$^{-1}$ (1.56 mM Ni(II)), and 1638.2 cm$^{-1}$ (2.56 mM Ni(II)). The number of smoothing points for the second derivate was 13. The gray spectrum shows the difference between the second derivatives of the spectra with zero Ni(II) and with 2.56 mM Ni(II). The colors of the other spectra match those of the CD spectra in Fig. 3 in the main manuscript to facilitate the comparison.

**The effect of Ni(II) ions on the secondary structure of Aβ peptides**

From Fig. 3 in the main manuscript, it can be seen that addition of Ni(II) ions induces structural transitions under some conditions. The CD measurements of titrations with Ni(II) acetate show clear transitions with isodichroic points for 10 μM $A\beta_{40}$ wt (Fig. 3D), and for 10 μM Aβ(4-40) peptide (Fig. 4F), both in 20 mM sodium phosphate buffer, pH 7.3 at 25 °C. Fig. S1 shows the difference spectra for these two titrations, created by subtracting the spectra with no added Ni(II) acetate from the spectra with 256 µM Ni(II) acetate. For both peptides the difference spectra correspond to beta sheet structures (Greenfield & Fasman, 1969), indicating that the structural changes are from random coil (the initial structures) to β-sheet.

The structure-altering effects of Ni(II) ions were investigated also by IR spectroscopy. Fig. S2 shows the second derivatives of the IR absorbance in the amide I range for synthetic $A\beta_{40}$ wt peptides at pD 12 (10 mM NaOD) and different concentrations of Ni(II) acetate. All spectra are dominated by a random coil band near 1639 $cm^{-1}$. With increasing Ni(II) concentration, the low wavenumber side of this band shifts to lower wavenumbers, and at the highest Ni(II) concentration a clear shoulder at 1633 $cm^{-1}$ can be observed. This spectral position is characteristic of β-sheets. The amplitude near 1680 $cm^{-1}$ is reduced at the highest Ni(II) concentration, which is in line with the presence of the high wavenumber band of antiparallel β-sheets. In accord with these observations, a difference second derivative spectrum (2.56 mM Ni(II) minus no Ni(II), gray line in Fig. S2) shows two negative bands at 1680 and 1629 $cm^{-1}$, indicating antiparallel β-sheet formation at high Ni(II) concentrations. The positive band at 1644 $cm^{-1}$ corresponds to the associated loss of random coil structure.

**Sodium dodecyl sulfate polyacrylamide gel electrophoresis (SDS-PAGE) analysis of SDS-stabilized $A\beta_{42}$ oligomers**

Formation of SDS-stabilized $A\beta_{42}$ oligomers in presence of Ni(II) ions was studied by SDS-PAGE, using both synthetic (Fig. S3A) and recombinant (Fig. S3B) $A\beta_{42}$ peptides. Due to

the high concentrations of SDS in the sample buffer, the Aβ oligomer distribution becomes modified, and the method is therefore not reliable on its own when used for studies of Aβ oligomers (Pujol-Pina *et al*, 2015). However, it is still possible to investigate the effects of different factors on oligomer formation, by comparing the size distribution pattern of the produced oligomers on SDS denaturing gels.

Fig. S3A shows the results of SDS-PAGE separation of synthetic SDS-stabilized oligomers exposed to different Ni(II) concentrations. Bands corresponding to monomers and trimers/tetramers are resolved for $A\beta O_{0.2\%SDS}$ . For $A\beta O_{0.05\%SDS}$ , bands of 32/40 kD are also observed. In presence of 100 μM and 500 μM Ni(II) ions, an additional weak band just below 20 kDa appears right above the tetrameric $A\beta_{42}$ band near 16 kDa, for both types of $A\beta_{42}$ oligomers.

Fig. S3B presents respective results for a recombinant $A\beta_{42}$ peptide. The Ni(II) ion effects are generally similar to those obtained for the synthetic peptide (Fig. S3A), although the band pattern is clearly different and in general narrower bands are observed on the gel with the recombinant $A\beta_{42}$. Thus, various bands of different intensities are detected for the monomeric and oligomeric samples. Monomeric solution mainly produces bands corresponding to monomer, dimer, and tetramer. For both types of oligomers, bands with sizes between 15-20 kDa (i.e., tetramers) dominate the corresponding lanes, while bands of larger molecular weights (particularly between 25-75 kDa) are also observed. For oligomeric samples, bands with sizes of monomers to trimers are much less intense on the gel. As the concentration of Ni(II) ions in the oligomerizaton solutions increases, the oligomer size distribution on the SDS-PAGE gel shifts towards molecular weights that are both smaller and larger than the major bands observed for SDS-stabilized oligomers. This is particularly evident in Fig. S3B. This can be interpreted as general interference of the Ni(II) ions with the formation of homogeneous SDS-stabilized $A\beta_{42}$ oligomers, thereby shifting the oligomerization process towards the formation of a more diverse population of oligomeric species. Such oligomers are possibly less resistant to the effects of high SDS

concentrations in the SDS-PAGE sample buffer, and may therefore produce larger amounts of different SDS-induced aggregation "by-products" after exposure to 2% SDS in the sample buffer (Bitan *et al*, 2005). Another explanation is that the effects of the high SDS concentration from the sample buffer on the oligomer distribution are modulated by the Ni(II) ions. The second derivatives of the IR absorbance of these oligomers are presented in Fig. 8 of the main text. In addition, Fig. S4 shows the absorbance spectra after solvent and baseline subtraction.

**Materials and methods: SDS polyacrylamide gel electrophoresis of A$\beta_{42}$ oligomers**

The A$\beta_{42}$ oligomer samples prepared with 0–500 µM Ni(II) acetate, as described in the materials section, were studied by Sodium Dodecyl Sulfate PolyAcrylamide Gel Electrophoresis (SDS-PAGE). The samples were incubated with the sample buffer containing 2% (w/v) of SDS at room temperature for 5 minutes without heating, loaded on Mini-PROTEAX TGX precast gels (Bio-Rad, USA), and used together with SDS-PAGE running buffer and an electrophoresis system (Bio-Rad, USA). The Precision Plus Protein Dual Color Standard (Bio-Rad, USA) was used as the protein molecular weight marker. The samples were run at 4 °C for 90 minutes and stained with the Pierce Silver Staining Kit (ThermoFisher Scientific, USA).

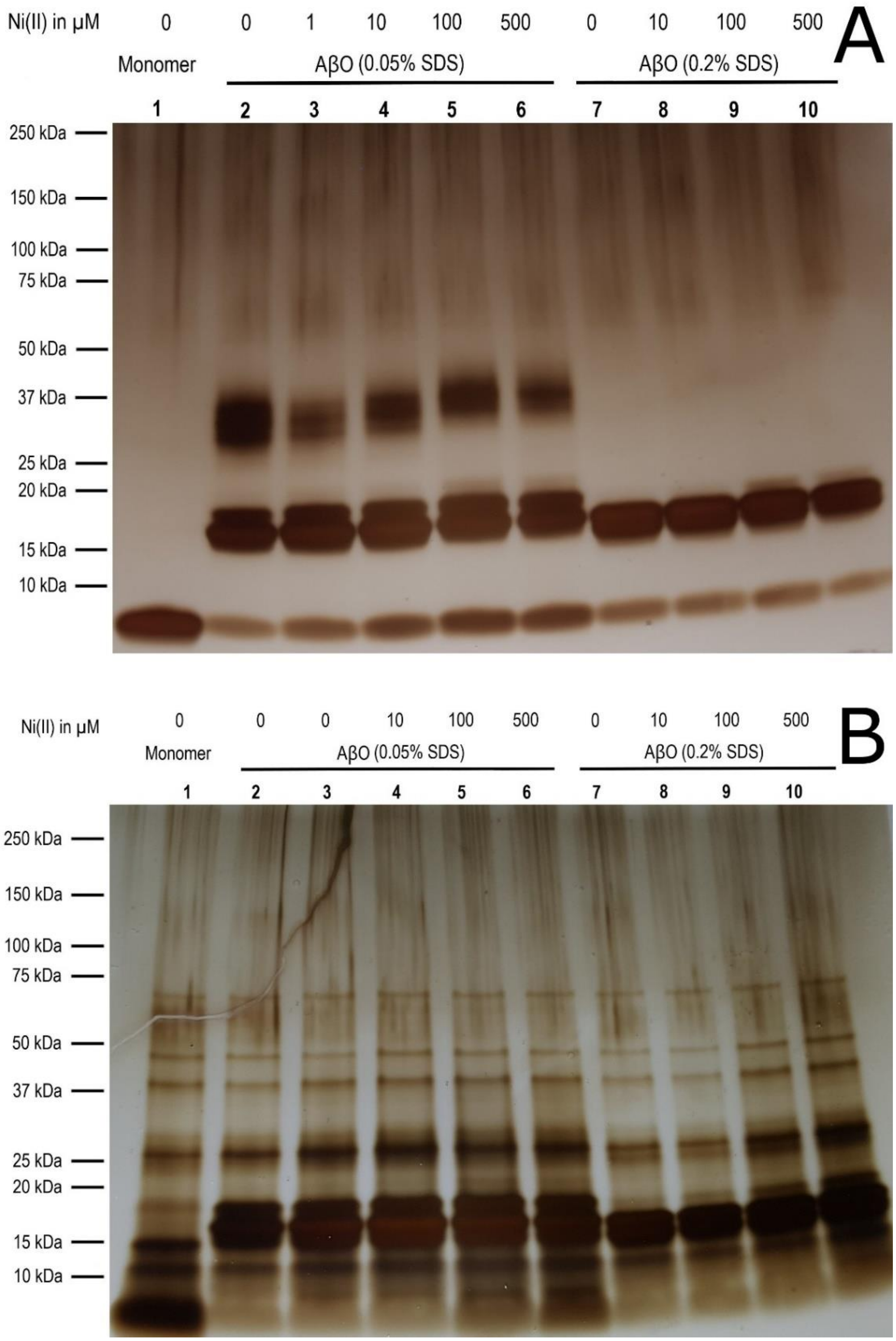


**Fig. S3**. SDS-PAGE analysis of the effects of Ni(II) ions on the formation of SDS-stabilized A$\beta_{42}$ oligomers created by either synthetic (A) or recombinant (B) A$\beta_{42}$ peptides. Lanes: 1. Monomers; 2-6. A$\beta$O$_{0.05\%SDS}$; 7-10. A$\beta$O$_{0.2\%SDS}$ with the Ni(II) concentrations indicated in the figure.

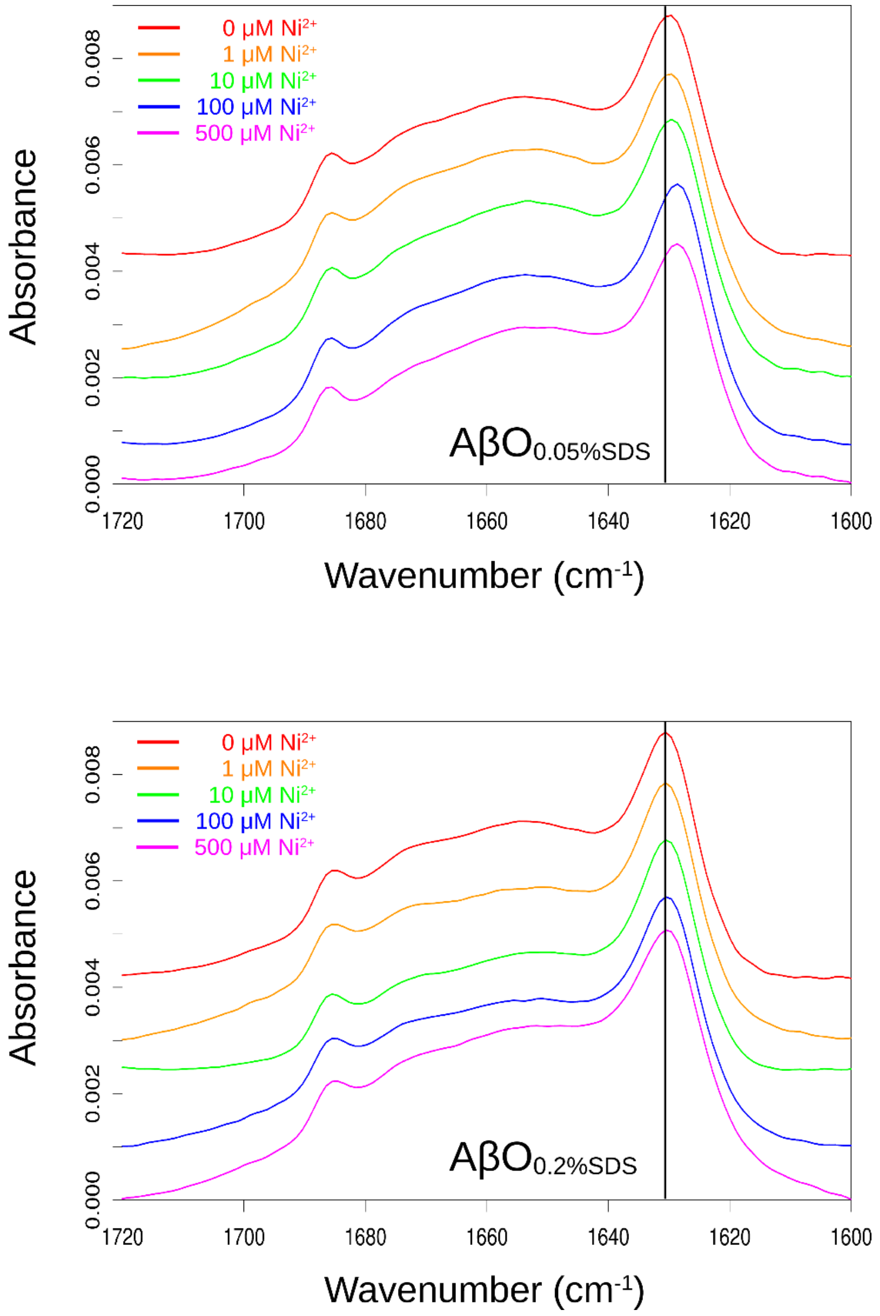


**Fig. S4**. Infrared absorbance spectra of SDS-stabilized oligomers after subtraction of a spectrum of the respective SDS-containing solvent, subtraction of a polynomial baseline generated at 4 points (1760, 1720, 1600, 1590 cm−1) and subtraction of residual water vapor bands, when necessary. The color code matches that of Fig. 8 of the main text. The vertical line in both panels indicates the spectral position of the main β-sheet band of the oligomers prepared in 0.2% SDS without Ni(II) ions.

## References

Bitan, G. (2006) Structural study of metastable amyloidogenic protein oligomers by photo-induced cross-linking of unmodified proteins. Methods Enzymol. 413, 217−236.

Greenfield, N., and Fasman, G. D. (1969) Computed circular dichroism spectra for the evaluation of protein conformation. Biochemistry 8(10), 4108-4116.

Pujol-Pina, R., Vilaprinyó-Pascual, S., Mazzucato, R., Arcella, A., Vilaseca, M., Orozco, M., and Carulla, N. (2015) SDS-PAGE analysis of Aβ oligomers is disserving research into Alzheimer's disease: appealing for ESI-IM-MS. Sci. Rep. 5, 14809.
